# Functional profiling of gut microbial and immune responses toward different types of dietary fiber: a step toward personalized dietary interventions

**DOI:** 10.1080/19490976.2023.2274127

**Published:** 2023-11-09

**Authors:** Jian Tan, Rosilene V. Ribeiro, Christopher Barker, Claire Daien, Erick De Abreu Silveira, Andrew Holmes, Ralph Nanan, Stephen J. Simpson, Laurence Macia

**Affiliations:** a Charles Perkins Centre, The University of Sydney, Sydney, Australia; b School of Medical Sciences, Faculty of Medicine and Health, The University of Sydney, Sydney, Australia; c Centre for Education and Research on Ageing and Alzheimer’s Institute, Concord Hospital, University of Sydney, Sydney, Australia; d School of Life and Environmental Sciences, University of Sydney, Sydney, New South Wales, Australia; e Rheumatology, teaching hospital of Montpellier and University of Montpellier, Montpellier, France; f Inserm U1046, CNRS UMR 9214, Physiologie et Médecine Expérimentale du Cœur et des Muscles, (PhyMedExp), Montpellier, France; g Sydney Medical School and Charles Perkins Centre Nepean, The University of Sydney, Sydney, Australia; h Sydney Cytometry, The University of Sydney and The Centenary Institute, Sydney, Australia

**Keywords:** Dietary intervention, dietary fiber, fiber supplement, inulin, gut microbiome, personalized nutrition, short-chain fatty acid, immune profiling

## Abstract

Dietary fiber plays a crucial role in maintaining gut and overall health. The objective of this study was to investigate whether different types of dietary fiber elicited specific changes in gut microbiota composition and the production of short-chain fatty acids. To test this, a longitudinal crossover study design was employed, in which healthy adult women consumed three distinct dietary fiber supplements: Inulin (fructo-oligosaccharide), Vitafiber (isomalto-oligosaccharide), and Fibremax (mixture of different fiber) during a one-week intervention period, followed by a 2-week washout period. A total of 15 g of soluble fiber was consumed daily for each supplement. Samples were collected before and after each intervention to analyze the composition of the gut microbiota by 16S rRNA sequencing and fecal levels of short-chain fatty acids measured using nuclear magnetic resonance. Phenotypic changes in peripheral blood mononuclear cells were studied in subsets of participants with higher SCFA levels post-intervention using spectral flow cytometry. The results revealed substantial stability and resilience of the overall gut bacterial community toward fiber-induced changes. However, each supplement had specific effects on gut bacterial alpha and beta diversity, SCFA production, and immune changes. Inulin consistently exerted the most pronounced effect across individuals and certain taxa were identified as potential indicators of SCFA production in response to inulin supplementation. This distinguishing feature was not observed for the other fiber supplements. Further large-scale studies are required to confirm these findings. Overall, our study implies that personalized dietary fiber intervention could be tailored to promote the growth of beneficial bacteria to maximize SCFA production and associated health benefits.

## Introduction

Over the past few decades, the prevalence of non-communicable diseases in Western societies has risen dramatically. Environmental exposures, specifically the consumption of a diet low in dietary fiber is accepted as a significant risk factor. Low dietary fiber intake is associated with increased risk of all-cause mortality in humans,^1,2^ while preclinical studies have demonstrated that high dietary fiber intake protected against colitis, asthma, food allergies, arthritis, experimental autoimmune encephalitis, colorectal cancer, type 1 diabetes, and influenza infection.^3–10^ Consequently, the potential of fiber supplements to be utilized for disease prevention and/or intervention is an attractive strategy.^11^

Dietary fiber is a loosely defined term that refers to complex carbohydrates that are resistant to host digestion. Dietary fiber is utilized by gut bacteria as an energy source, and as prebiotics, they promote the growth of beneficial bacteria such as *Bifidobacteria* and *Lactobacilli*. The fermentation of dietary fiber by gut bacteria leads to the release of metabolites as by-products, particularly the short-chain fatty acids (SCFA) acetate, propionate, and butyrate.^12^ Butyrate is the major source of energy for colonocytes and maintains an anaerobic environment optimal for the survival of beneficial bacteria in the lumen.^13^ Butyrate and propionate are mostly metabolized in the liver, whereas acetate reaches the circulation and can exert systemic effects.^12^ There is considerable evidence from preclinical and clinical studies that SCFA support many of the health benefits of dietary fiber on the host. For example, daily treatment with propionate restored immune balance in multiple sclerosis patients and reduced their clinical symptoms,^14^ and acetate and butyrate have been shown to lower blood pressure in hypertensive patients.^15^

Furthermore, detrimental changes in gut microbiota composition, or dysbiosis, have been reported in most non-communicable diseases. Although direct causality between dysbiosis and disease is yet to be established, preclinical models have confirmed that changes in gut microbiota composition can contribute to the development of these diseases. Mice recolonized with dysbiotic microbiota isolated from patients’ stool develop exacerbated disease in models of allergies, autoimmunity, and during cancer therapy^16–18^. Thus, restoring gut microbiota composition and function, particularly by supporting optimal SCFA production through dietary fiber consumption, offers an attractive therapeutic approach.

There are conflicting results from clinical trials testing the impact of the dietary fiber supplement inulin and/or fructo-oligosaccharide on disease with either improvement or no effects.^19–23^ The reasons why certain individuals do not respond to inulin are unknown but is likely due to differences in their gut microbiota composition.^24–26^ Dietary fiber is composed of structurally diverse classes of polysaccharides, varying in their monosaccharide composition and chemical bonds^27^. As such, the repertoire of enzymes needed to break down these polysaccharides, such as carbohydrate-active enzymes, can differ widely between types of fiber. These carbohydrate-degrading enzymes are broadly dispersed across microbial strains.^28^ Hence, gut microbiota comprising distinct collections of strains will exhibit different responses to a given type of fiber due to differences in the enzymes they collectively possess. This was highlighted in a study by Kovatcheva-Datchary *et al*., which showed that the consumption of bread enriched in dietary fiber improved postprandial glucose metabolism only in participants with a microbiota characterized by an elevated *Prevotella/Bacteroides* ratio.^29^ A landmark study demonstrated that individuals’ specific postprandial blood glucose responses to varying foods could be predicted by their preexisting gut microbial communities.^30^ These studies raise the possibility of personalized nutrition, such as offering individuals tailored dietary fiber interventions to yield maximal SCFA production.

In this study, we examined individual responses to three different dietary fiber supplements and tested the hypothesis that an individual’s SCFA profile in response to a given dietary fiber can be predicted based on their gut microbiota composition. This was achieved through a cross-over intervention study to test the response of given individuals to the three different fiber supplements. We showed that although the gut microbiota was largely unchanged, there were supplement-specific effect between longitudinal samples of individuals on specific bacterial taxa, SCFA production, and the immune system. We also identified taxa that may be predictive of an individual’s likelihood of responding to inulin supplementation; however, a larger-scale study is required to confirm our findings.

## Results

### Impact of different fibre supplements on clinical characteristics in healthy women

**Figure 1.**
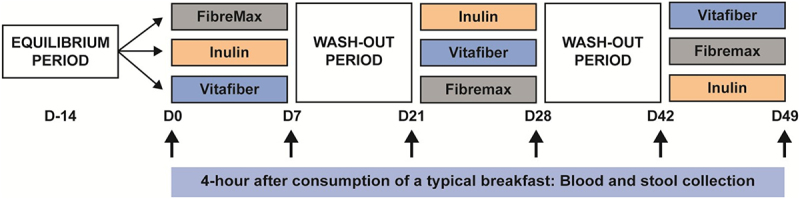
Cross-over study design in which the participants were asked to consume one of three dietary fiber supplements (Fibremax, Inulin or Vitafiber) daily for 1 week, with a two-week washout period between supplements. Prior to each dietary fiber intervention, and throughout the study, participants were asked to consume a standardized diet which excludes fiber-rich foods including beans, high-fiber cereals and bars, dietary supplements and antibiotics. Before and after each intervention, stool and blood were collected 4 hours following the consumption of a typical breakfast.

Thirty-four healthy females were recruited for a crossover study, with all participants asked to intake three different fiber supplements for a period of 1 week per supplement, with a 2-week washout period between supplements (Figure 1). The three commercially available fiber supplement products used were Inulin (100% fructo-oligosaccharide), Vitafiber (100% Isomalto-oligosaccharide; IMO), and Fibremax (a mix of soluble and insoluble fiber consisting of chicory root extract, psyllium husk, soy fiber, oat bran and pectin). We chose structurally distinct dietary fibers: Inulin is a polymer of fructose with β-(2,1) glycosidic bonds and Vitafiber a polymer of glucose with α-D-(1,6)-linkages and Fibremax which contains a diverse mix of dietary fiber. These dietary fibers are commonly used in the food industry and as supplements,^31^ and inulin (including FOS) are approved by FDA as a dietary fiber^32^ and have been used in clinical trials to tests its effect on various conditions such as obesity and asthma.^19,33^

Of those enrolled, 28 participants completed the study. The median age at screening interview was 37 (IQR 15.25) years and BMI was 22.1 (IQR 4.1) kg/m^2^ (Table 1). Reasons for withdrawing from the study included pregnancy (*n* = 1), intolerance to supplements (*n* = 2), inability to attend clinical assessments due to injury (*n* = 1), or lack of time (*n* = 2). Overall compliance with fiber supplementation was 93.1% for Fibremax, 96% for Inulin, and 95.6% for Vitafiber (calculated as the number of doses reported/total number of doses prescribed for each supplement). Self-reported symptoms in response to each supplement (bloating or gas) during the 7-day of intervention was 54.5% for Fibremax, 48% for Inulin and 25% for Vitafiber. There was one report of diarrhea for both Fibremax and Inulin but none for Vitafiber. Baseline and intervention dietary fiber (excluding supplements), energy, protein, fat, and carbohydrate intakes were similar across all groups (Table S1).

**Table 1.** Baseline characteristics of participants who completed the study and for those used in downstream microbiota analysis. Data represented as median (IQR).

Characteristics	All participant (*n* = 28)	Participants used for microbiota analysis (*n* = 24)
Age (years, IQR)	37.0 (15.25)	37.0 (16.25)
Sex (number, %)		
Female	28 (100)	24 (100)
Male	0 (0)	0 (0)
Weight (kg, IQR)	61.65 (12.5)	63.0 (12.5)
BMI (BMI, IQR)	22.1 (4.10)	22.1 (4.17)

Body weight, waist and hip circumference, waist-to-hip ratio, and systolic and diastolic blood pressure were measured before and after each dietary fiber intervention (Table 2). There was no major impact on all measured clinical characteristics, although waist circumference was significantly increased with the supplement Fibremax (median +1.0 cm [IQR −0.5 to 2.13], *p* =.014) and decreased with the supplement Vitafiber (median −0.5 cm [IQR −2.13 to 1.5], *p* =.024). After Inulin supplementation, we observed an increase in hip circumference (median +1.0 cm [IQR −1 to 2], *p* =.012) while Vitafiber supplementation increased diastolic blood pressure (median +2.5 mmHg [IQR −0.63 to 6.5], *p* =.017).

**Table 2.** Change in characteristics following intervention.

	Change, median (IQR)	t value	p value
Weight (kg)			
Fibremax	0.05 (−0.4, 0.4)	0.78	0.437
Inulin	0 (−0.7, 0.5)	−1.02	0.312
Vitafiber	0.05 (−0.5, 0.68)	−0.31	0.753
Waist circumference (cm)			
Fibremax	1 (−0.5, 2.13)	2.48	*0.014
Inulin	0 (−2.5, 1)	−0.87	0.387
Vitafiber	−0.5 (−2.13, 1.5)	−2.27	*0.024
Hip circumference (cm)			
Fibremax	−0.5 (−1, 1.13)	1.85	0.066
Inulin	1 (−1, 2)	−2.53	*0.012
Vitafiber	0.75 (−1.5, 1)	−0.96	0.339
Hip to Waist ratio			
Fibremax	−0.016 (−0.044, 0.0106)	−0.404	0.687
Inulin	−0.018 (−0.081, 0.005)	−0.177	0.860
Vitafiber	0.0171 (−0.029, 0.038)	0.197	0.844
Systolic blood pressure (mmHg)			
Fibremax	−3 (−5.38, 2.25)	−0.22	0.827
Inulin	−2 (−10, 4.5)	−0.52	0.603
Vitafiber	3.5 (−1.25, 6.25)	0.97	0.333
Diastolic blood pressure (mmHg)			
Fibremax	−2 (−5.5, 0.88)	−1.79	0.075
Inulin	−1 (−4.5, 3)	0.8	0.426
Vitafiber	2.5 (−0.63, 6.5)	2.41	*0.017

### Gut microbiota alteration in response to fibre supplements is constrained by individual characteristics

We assessed whether the 1-week intervention with the three fiber supplements had a discernible impact on gut microbial communities. To do this, we performed 16S rRNA sequencing from pre- and post-intervention stool samples from participants in which stool samples were collected from all time points (*n* = 24 out of 28 and baseline characteristics from this cohort is presented in Table 1). Sequencing output generated sufficient reads from each sample to capture all amplicon sequence variants (ASV) (Fig. S1A), and no significant differences in reads per sample between fiber supplements, either pre- or post-intervention, were observed (Fig. S1B).

**Figure 2.**
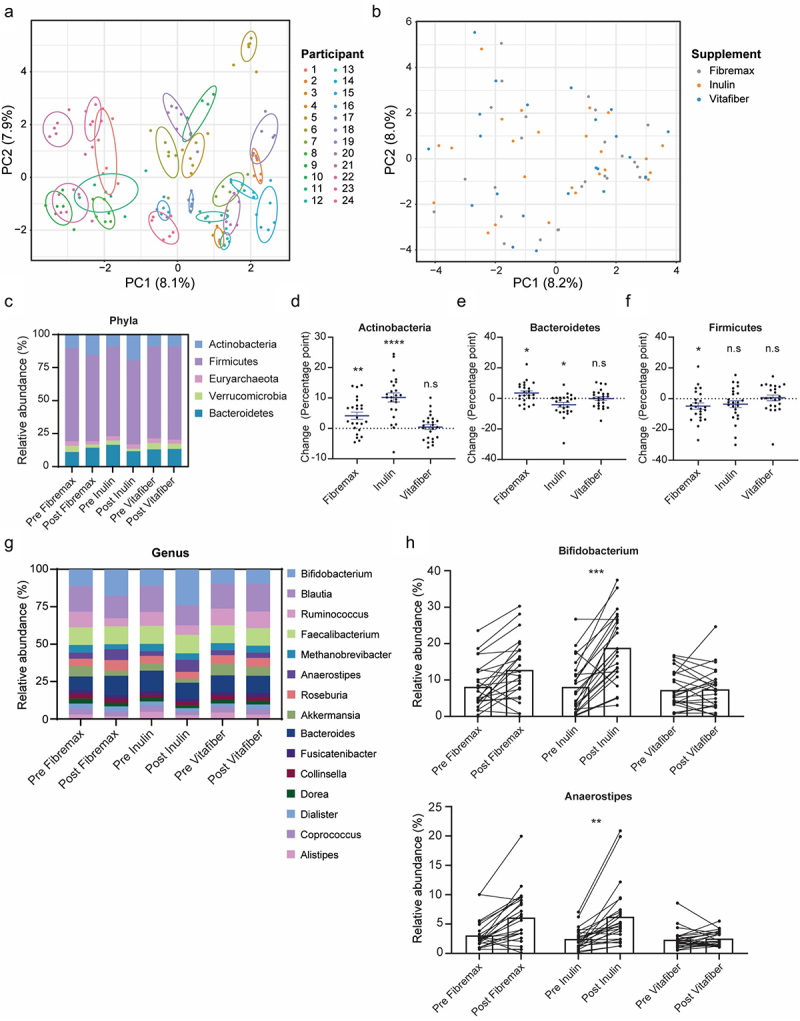
Stools were collected before and one-week following the consumption of either Fibremax, Inulin or Vitafiber and 16S rRNA gene sequencing was performed to analyze gut microbiota composition. (a) differences in the overall microbiota communities of all participants and timepoints were analyzed by principal component analysis (PCA) of Aitchison’s distance. Colour represents unique individuals, and each cluster of individuals were statistically significantly (*p* <.001) from all other cluster of individuals as determined by Kolmogorov Smirnov test of within vs. between cluster distributions of Aitchison distance. (b) differences in the overall microbiota communities of post-intervention samples were analyzed by principal component analysis (PCA) of Aitchison’s distance. Colour represents fiber supplements, and overall post-microbiome supplement were not significantly different between groups as determined by PERMANOVA (Inulin vs. Vitafiber *p* > 0.99; Inulin vs. Fibremax *p* > .99; Vitafiber vs. Fibremax *p* =.9998). (c) relative abundance of bacteria in the stool at the phylum level (top 5 shown). (d-f) the absolute percentage change of (d) *Actinobacteria*, (e) *Bacteroidetes* and (f) *Firmicutes* following intervention with either Fibremax, Inulin or Vitafiber. *p <.05, **p <.01 and ****p <.0001 as determined by paired t-test between before and after intervention for each individual supplement. Data represented as mean ± SEM. (g) relative abundance of bacteria in the stool at the genus level (top 15 shown). (h) before/after plot of the relative abundance of *Bifidobacterium* (top) and *Anaerostipes* (bottom) for Fibremax, Inulin and Vitafiber. **p <.01 and ***p <.001 as determined by paired t-test between before and after intervention for each individual supplement.

A global analysis of all samples by non-supervised clustering of principal component analysis (PCA) of Aitchison’s distance revealed that the principal driver of microbiota composition variation was the individual participant (Figure 2a). No evidence of a generic response to fiber was found, with no significant clustering of post-supplement gut microbiota composition (Figure 2b). Similarly, there was no significant clustering between pre- and post-supplementation microbiota for any of the three supplements (Fig. S1C). Altogether, one week of dietary fiber supplementation, regardless of the type of supplement, did not alter the overall gut microbiota composition to become significantly more similar.

### Fibre supplementation induced signature changes to the gut microbiota composition

While none of the supplements altered the overall gut microbiota composition as measured using non-supervised clustering by PCA of Aitchison’s distance, we next investigated whether the consumption of fiber supplements led to more specific changes in the gut microbiota. Consistent changes to the relative abundance of taxa at the phylum level were observed in response to a specific fiber supplementation, regardless of the individuals’ pre-supplement microbiome (Figure 2c). Notably, *Actinobacteria* were significantly increased following Inulin (23/24 patients, *p*=<0.0001) and Fibremax (18/24 patients, p = .0017) supplementation (Figure 2d). In contrast, *Bacteroidetes* level was significantly increased by Fibremax (17/24 patients, p = 0.0172) and decreased by Inulin (15/24 patients, p = .0112) (Figure 2e), and *Firmicutes* decreased following Fibremax intervention (16/24 patients, p = .0286) (Figure 2f). *Euryarchaeota*, *Verrucomicrobia* and *Proteobacteria* were not affected by any supplement (Fig. S2A), and Vitafiber supplementation induced no changes to any phyla (Figure 2d–f, Fig. S2A). A summary of these changes is provided in Table 3. At the genus level (Figure 2g), differential abundance analysis of the top 30 genera (encapsulating 95% of total taxa) between pre- and post-supplement microbiota revealed that only Inulin induced a signature change, with a significant enrichment of *Bifidobacterium* (effect size = 1.47, p = .0002) and *Anaerostipes* (effect size = 1.25, p = 0.0017) after one week of supplementation (Figure 2h). When comparing post-supplement microbiota, Inulin had a significant enrichment in *Bifidobacterium* (effect size = 1.12, p = 0.0045) and *Anaerostipes* (effect size = 0.75, p =.037) over Vitafiber, but not over Fibremax.

**Table 3.** Supplement induced changes at the phyla level. Data represented as median (IQR). P-value represents paired t-test between baseline and intervention relative abundance value.

		Actinobacteria	Firmicutes	Euryarchaeota	Verrucomicrobia	Bacteroidetes	Proteobacteria
Fibremax	Baseline	8.61 (5.12, 12.75)	72.77 (64.38, 78.28)	0.88 (0, 6.42)	3.35 (0.37, 6.29)	10.82 (8.25, 14.59)	0.67 (0.26, 1.67)
	Intervention	7.07 (18.92, 11.85)	60.44 (73.63, 13.19)	0 (5.92, 5.92)	0.15 (3.02, 2.87)	9.36 (20.88, 11.52)	0.24 (0.91, 0.67)
	Change	2.96 (−0.35, 7.54)	−5.99 (−11.71, 2.06)	0 (−1.76, 0)	−1.74 (−4.28, 0.49)	2.16 (−0.93, 8.49)	−0.26 (−0.98, 0.16)
	p value	.0017	.0286	.1372	.2468	.0172	.0605
Inulin	Baseline	7.39 (3.49, 13.11)	67.8 (64.07, 73.61)	0.66 (0, 5.01)	0.82 (0.22, 6.96)	14.77 (11.29, 19.06)	0.77 (0.49, 1.45)
	Intervention	17.36 (12.68, 26.36)	67.57 (57.16, 70.54)	0.22 (0, 4.55)	1.31 (0.38, 3.31)	10.87 (7.23, 14.45)	0.35 (0.18, 0.85)
	Change	10.56 (6.19, 14.97)	−2.44 (−10.53, 4.38)	0 (−0.93, 0)	−0.05 (−3.11, 0.41)	−1.99 (−8.43, 0.4)	−0.3 (−0.82, 0.15)
	p value	<.0001	.1306	.8703	.0724	.0112	.0857
Vitafiber	Baseline	6.9 (4.39, 13.83)	69.91 (65.53, 76.18)	1.43 (0, 7.48)	2.19 (0.42, 4.99)	12.71 (10, 17.55)	0.81 (0.33, 1.34)
	Intervention	8.19 (4.28, 12.03)	71.67 (68.55, 74.76)	0.92 (0, 6.51)	2.86 (0.29, 4.1)	12.26 (8.7, 17.98)	0.61 (0.38, 1.85)
	Change	0.67 (−3.38, 2.99)	0.32 (−4.75, 8.05)	0 (−0.97, 0)	0.07 (−1.88, 1.54)	0.32 (−5.04, 3.48)	0.17 (−0.54, 0.82)
	p value	.6267	.7742	.1626	.772	.9318	.2145

We then next asked whether each intervention induced a specific bacterial taxonomic signature at the ASV level, using supervised machine learning (Sparse partial least squares discriminant analysis; sPLS-DA) to build models classifying samples in accordance with post-intervention microbiota. This approach considers the overall complexity of the gut microbiota, rather than by comparing individual taxa. Post-supplementation samples could be discriminated against each other with an accuracy of 77.8% (Fig. S2B) (*p* =.02). Within the model, an Inulin supplemented microbiota had the most distinguishing features, with ASVs assigned as *Anaerostipes hadrus*, *Holdemanella spp*., and *Mogibacterium spp*. having the greatest contribution to the separation of the different post-supplementation microbiota (Fig. S2C and Table S2). In contrast, ASVs corresponding to *Blautia*, *Clostridium IV* species, and others were associated with Fibremax, while only *Blautia faecis* was marginally predictive of a Vitafiber supplemented microbiome (Fig. S2C and Table S2).

Together, we show that the composition of the gut microbiota can be selectively influenced by just one week of fiber supplementation. This effect was fiber specific with a significant increase in *Bifidobacteria*, a group of bacteria known for their ability to utilize dietary fiber, following the consumption of Inulin and Fibremax in the majority of participants. Fiber supplement-induced changes were substantial enough that post-supplement signatures could be reliably differentiated using a machine learning approach.

### The effect of fibre supplementation on gut microbiota composition can be effectively eliminated with a two-week washout period.

**Figure 3.**
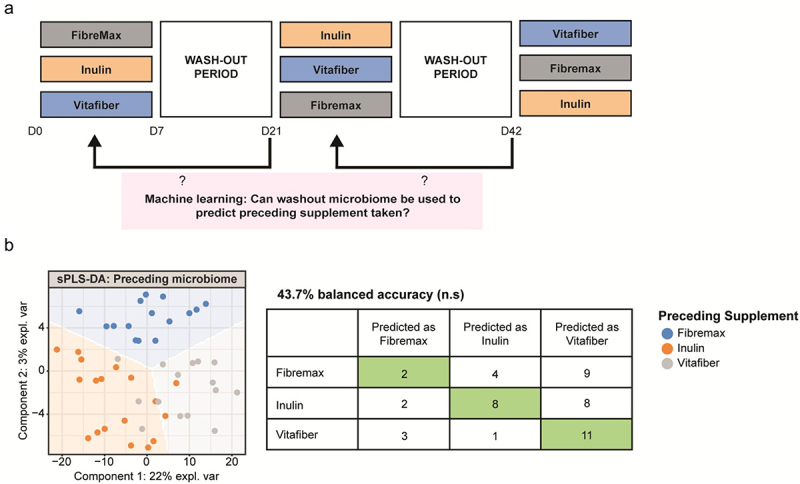
Stools were collected before and one-week following consumption of either Fibremax, Inulin or Vitafiber and 16S rRNA gene sequencing was performed to analyze gut microbiota composition. (a) schematic highlighting the supervised machine learning approach to determine if a two-week washout period is sufficient to reset the gut microbiome (b) scatter plot of sPLS-DA analysis illustrating the predicted gut microbiome composition of pre-supplement samples (left) and confusion matrix outlining the performance of the prediction model (right) and loading plots indicating the ASV contributing to the sPLS-DA model (left).

To confirm whether a two-week washout period was sufficient, we applied supervised machine learning to build a model that classified post-washout (pre-supplement) samples in accordance with the preceding supplement. Pre-supplement samples from the first batch were excluded because there was no preceding intervention (Figure 3a). The resultant model correctly classified only 43.7% (vs. 33% for random guessing) of samples based on the preceding supplement (Figure 3b), which was not statistically significant (*p* =.22), indicating that preceding supplements did not induce any distinguishing changes in microbiomes that persisted past the washout period.

Thus, the 2 week-long washout period between the 1-week supplement interventions is sufficient for fiber supplement-induced effects on the gut microbiome to be lost. It also suggests that dietary fiber supplementation induced short-term and reversible alterations to gut microbiota.

### Inulin and fibremax but not vitafiber supplementation reduced gut microbiota diversity.

**Figure 4.**
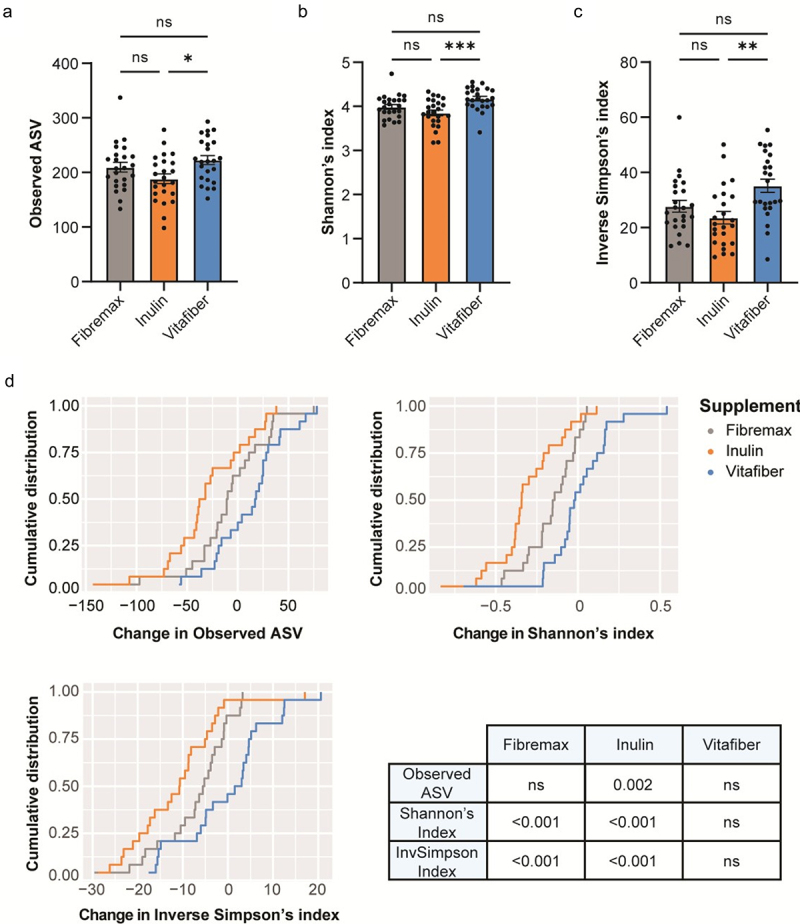
Stool were collected before and one-week following consumption of either Fibremax, Inulin or Vitafiber and 16S rRNA gene sequencing was performed to analyze gut microbiota composition. (a-c) alpha diversity measures of post-supplement gut microbiome as measured by (a) observed ASV (b) Shannon’s diversity index and (c) Inverse Simpson’s diversity index. Data represented as mean ± SEM and *p < 0.05, **p < 0.01 and ***p < 0.001 as determined by ordinary one-way ANOVA with Tukey’s multiple comparison. (d) cumulative distribution function (CDF) plot showing the cumulative change in diversity measures across all participants for each of the three dietary fiber supplements. Table shows the statistical output detailing whether the differences in a given alpha diversity measure significantly differs from zero based on paired Wilcoxon signed-rank test.

Different fiber supplements had differential effects on gut microbial community alpha diversity metrics. When comparing post-supplement microbiota, we found that inulin induced a significant decrease in bacterial richness (Observed ASV), Shannon’s index, and the Inverse Simpson’s index compared to other interventions (Figure 4a–c). Importantly, no such differences were evident in pre-intervention samples (Fig. S3A), confirming that a 2-week washout period was sufficient to normalize the gut microbiota of each participant.

When comparing changes induced by specific fiber interventions, we found that Inulin had the most consistent effect across individuals on alpha diversity measures, with approximately 75% of the participants showing a decrease in observed ASV (Figure 4d), while Fibremax had an intermediate impact and Vitafiber had no impact. Overall, Inulin significantly decreased post-intervention bacterial richness (Observed ASV), Shannon’s and Inverse Simpson’s diversity indices relative to pre-intervention (Figure 4d and Fig. S3B). Fibremax decreased Shannon’s and Inverse Simpson’s diversity indices relative to pre-intervention values (Figure 4d and Fig. S3C), while Vitafiber had no impact on gut microbiota diversity across all measures (Figure 4d. and Fig. S3D).

The alpha diversity results support our previous observations that, while Inulin and Fibremax affected the gut microbiota, Vitafiber had little or no effect. To explore this, we used supervised machine learning to build a model to classify post-intervention microbiota (as done in Fig. S2B), but with the inclusion of baseline samples. In this model, Inulin and Fibremax could be differentiated with high accuracy, whereas the model could not distinguish between baseline samples and Vitafiber-supplemented gut microbiota (Fig. S3E). The inability of the sPLS-DA model in finding features that could differentiate between post-supplement Vitafiber and baseline microbiome suggests that Vitafiber supplementation does not have a prominent impact on gut microbiota composition.

Overall, both Inulin and Fibremax supplementation reduced gut microbiota alpha diversity, with inulin having the most significant effect. This is consistent with our previous analysis showing that Inulin has the greatest impact on the gut microbiota (Figure 2c–h).

### Short-chain fatty acid production in response to dietary fibre is individual and fibre-specific.

We next investigated how the different fiber supplements altered stool SCFA profiles, a proxy measure of the effectiveness of microbe-stimulation by fiber supplements, in study participants. When comparing the change in stool SCFA levels between pre- and post-intervention samples, there was no significant difference between any of the supplements (Fig. S4A). Consistent with these findings, post-intervention stool SCFA levels were similar across the three fiber interventions (Fig. S4B). These data indicate that, on average, there was no increase or decrease in specific SCFAs when comparing the entire study population.

**Figure 5.**
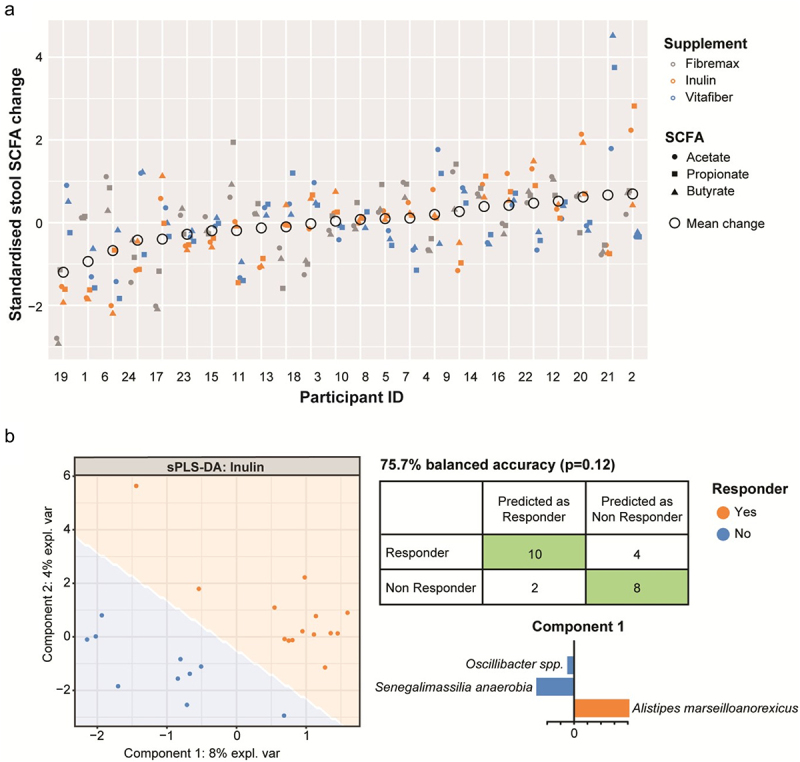
Stools were collected before and one-week following consumption of either Fibremax, Inulin or Vitafiber and stool SCFA concentrations were quantified by NMR. (a) standardized change in SCFA for each participant is shown. Circles represent the mean change in stool SCFA following intervention of each individual. (b) pre-supplement microbiome of responders (as determined by net increase of stool SCFA after dietary fiber supplementation) vs. non-responders were stratified, and pre-supplement features (taxa) that are positively or negatively associated with responders vs. non responders for Inulin were classified by supervised sPLS-DA analysis as illustrated by the scatter plot representing the two components of the model (left). Confusion matrix outlining the performance of the prediction model (right) and loading plots indicating the ASV contributing to the sPLS-DA model, with orange representing responders and blue representing non-responders. The length of the bar plot represents the importance of each ASV to the model (positive loading represents that the ASV is positively associated with either responders or non-responders while negative loading represents a negative association).

However, we found variation around zero change, consistent with fiber supplementation having personalized effects. To test this explicitly, we normalized SCFA changes (acetate, butyrate, and propionate) to lie on similar ranges of values, and then performed a non-parametric analysis of variance to test whether changes to SCFA were due to the participant irrespective of SCFA type or supplement; the supplement irrespective of SCFA type or participant; the interaction between participant and SCFA type or supplement; or the interaction between SCFA type and supplement. We found a statistically significant effect by participants (R^2^ = 0.23, *p* =.001), indicating that participants were differentially predisposed to SCFA level changes under fiber supplementation (Figure 5a). Furthermore, we found a significant effect of the interaction between participant and supplement type, meaning that changes in SCFA in individuals are supplement specific (R^2^ = 0.61, *p* =.001). We found no evidence of: 1) specific supplements affecting overall SCFA changes (irrespective of SCFA type or specific participant), 2) specific supplements on specific SCFA types, or 3) participants being differentially predisposed to changes in a specific SCFA type.

Plasma acetate, the prominent SCFA in blood, exhibited similar patterns to stool SCFA, with no significant change in plasma acetate levels between pre- and post-intervention samples (Fig. S4C), as well as when comparing post-intervention samples (Fig. S4D). There was a significant effect of the interaction between participant and supplement type (R^2^ = 0.228, *p* =.046), indicating that changes in plasma SCFA in individuals are also supplement specific (Fig. S4E).

Collectively, our data suggest that changes in SCFA levels in response to fiber supplementation depend on both the individual and the type of fiber supplement.

### Specific microbial taxa are associated with SCFA production in response to inulin supplementation

It is evident that the response to dietary fiber (related to SCFA) depends on the composition of the gut microbiota and the type of fiber supplement consumed. Thus, we next asked whether an individual’s unique gut microbiota composition would dictate their response to different fiber supplements. To do this, we first stratified individuals as either responders (increase in total stool SCFA concentration) or non-responders (no change or decrease in total stool SCFA concentration) for each supplement. Next, we performed supervised sPLS-DA analysis on pre-supplement microbiota to identify features (taxa) associated with either responders or non-responders to each fiber supplement. Using this approach, a satisfactory model for Fibremax and Vitafiber could not be produced, resulting in over-fitted models that included most of the taxa present and had poor predictive accuracy (Fig. S5A and Fig. S5B). However, Inulin could produce a satisfactory model that could predict whether an individual would respond to Inulin supplementation with 75.7% accuracy (Figure 5b), although this was not statistically significant (*p* =.12). Within this model, the butyrate producer *Alistipes marseilloanorexicus* was predictive of responders, whereas non-responders were characterized by lower *Senegalimassilia anaerobia* and *Oscillibacter spp* (Figure 5b and Table 4). No satisfactory models could be produced for any of the supplement when stratifying responders and non-responders based on plasma acetate levels (data not shown).

**Table 4.** Taxa (components) relating to sPLS-DA model of responders vs. non responders to Inulin.

ASV	Taxa ASV belongs to	Contribute to group	Comp	Loading
TACGGAGGATCCGAGCGTTATCCGGATTTATTGGGTTTAAAGGG TGCGTAGGCTGTTTTTTAAGTTAGAGGTGAAAGCTCGACGCTCA ACGTCGAAATTGCCTCTGATACTGAGAGACTAGAGTGTAGTTGC GGAAGGCGGAATGTGTGGTGTAGCGGTGAAATGCTTAGATATC ACACAGAACACCGATTGCGAAGGCAGCTTTCCAAGCTATTACTG ACGCTGAGGCACGAAAGCGTGGGGAGCGAACAGG	Alistipes marseilloanorexicus	Responders	1	0.822
TACGTATGGGGCGAGCGTTATCCGGATTCATTGGGCGTAAAGCGC GCGTAGGCGGAGCGCTAAGCGGGACCTCTAACCCGAGGGCTCAA CCCCCGGCCGGGTCCCGAACTGGCGCTCTCGAGTGCGGTAGGGGA GAGCGGAATTCCCGGTGTAGCGGTGGAATGCGCAGATATCGGGAA GAACACCGACGGCGAAGGCAGCTCTCTGGGCCGAAACTGACGCTG AGGCGCGAAAGCTGGGGGAGCGAACAGG	Senegalimassilia anaerobia	Non-responders	1	−0.560
TACGTAGGTGGCAAGCGTTGTCCGGATTTACTGGGTGTAAAGGGCG TGTAGCCGGGAAGGCAAGTCAGATGTGAAATCCACGGGCTTAACTC GTGAACTGCATTTGAAACTGTTTTTCTTGAGTATCGGAGAGGCAATC GGAATTCCTAGTGTAGCGGTGAAATGCGTAGATATTAGGAGGAACA CCAGTGGCGAAGGCGGATTGCTGGACGACAACTGACGGTGAGGCG CGAAAGCGTGGGGAGCAAACAGG	Oscillibacter	Non-responders	1	−0.101
TACGTATGGTGCAAGCGTTATCCGGATTTACTGGGTGTAAAGGGAG CGCAGGCGGTCTGGCAAGTCTGATGTGAAAGCCCGGGGCTCAACC CCGGGACTGCATTGGAAACTGTCAGACTAGAGTGTCGGAGAGGTA AGTGGAATTCCTAGTGTAGCGGTGAAATGCGTAGATATTAGGAGG AACACCAGTGGCGAAGGCGGCTTACTGGACGATAACTGACGCTGA GGCTCGAAAGCGTGGGGAGCAAACAGG	Roseburia	Responders	2	0.567
TACGTAGGGGGCAAGCGTTATCCGGATTTACTGGGTGTAAAGGGA GCGTAGACGGCAAGGCAAGTCTGAAGTGAAAGCCCGGTGCTTAAC GCCGGGACTGCTTTGGAAACTGTTTAGCTGGAGTGCCGGAGAGGT AAGCGGAATTCCTAGTGTAGCGGTGAAATGCGTAGATATTAGGAA GAACACCAGTGGCGAAGGCGGCTTACTGGACGGTAACTGACGTTG AGGCTCGAAAGCGTGGGGAGCAAACAGG	Lachnospiraceae	Non-responders	2	−0.551
TACGTAGGGGGCAAGCGTTATCCGGAATTATTGGGCGTAAAGAGT ACGTAGGTGGTTTTCTAAGCACGGGGTTTAAGGCAATGGCTTAACC ATTGTTCGCCTTGTGAACTGGAAGACTTGAGTGCAGGAGAGGAAA GCGGAATTCCTAGTGTAGCGGTGAAATGCGTAGATATTAGGAGGA ACACCAGTGGCGAAGGCGGCTTTCTGGACTGTAACTGACACTGAG GTACGAAAGCGTGGGGAGCAAACAGG	Clostridiales	Responders	2	0.471
TACGTAGGTGGCAAGCGTTGTCCGGATTTACTGGGTGTAAAGGGCG TGTAGGCGGAGCAGCAAGTCAGAAGTGAAATCTCTGGGCTCAACCC AGAAACTGCTTTTGAAACTGTTGCCCTTGAGTATCGGAGAGGCAGGC GGAATTCCTAGTGTAGCGGTGAAATGCGTAGATATTAGGAGGAACAC CAGTGGCGAAGGCGGCCTGCTGGACGACAACTGACGCTGAGGCGCG AAAGCGTGGGGAGCAAACAGG	Sporobacter	Responders	2	0.254
TACGTAGGTGGCGAGCGTTATCCGGATTTATTGGGCGTAAAGCGTCC GCAGCCGGTTTATTAAGTCTAGAATAAAAGCCTGGAGCTTAACTCCA GTTCGTTCTAGAAACTGATATACTTGAGTGTAGTAGAGGCAAATGGA ATTTCTAGTGTAGCGGTAAAATGCGTAGATATTAGAAGGAACACCAG TGGCGAAGGCGATTTGCTAGGCTATTACTGACGGTCAGGGACGAAAG CGTGGGGAGCAAATAGG	Firmicutes	Responders	2	0.163
TACGTAGGTGGCGAGCGTTGTCCGGAATTATTGGGCGTAAAGGGAGC GCAGGCGGGAGATCAAGTCTATCTTAAAAGTGCGGGGCTCAACCCCG TGAGGGGATGGAAACTGGTCTTCTTGAGTGCAGGAGAGGAAAGCGGA ATTCCTAGTGTAGCGGTGAAATGCGTAGATATTAGGAGGAACACCAGT GGCGAAGGCGGCTTTCTGGACTGTAACTGACGCTGAGGCTCGAAAGCG TGGGGAGCGAACAGG	Mitsuokella multacida	Responders	2	0.144
TACGTAGGTGGCAAGCGTTGTCCGGAATTATTGGGCGTAAAGGGCGCG CAGGTGGTTTCTTAAGTCTGTCTTAAAAGTGCGGGGCTTAACCCCGTGA GGGGACGGAAACTGGGAGACTAGAGTATCGGAGAGGAAAGCGGAATT CCTAGTGTAGCGGTGAAATGCGTAGATATTAGGAGGAACACCAGTGGC GAAAGCGGCTTTCTGGACGACAACTGACACTGAGGCGCGAAAGCCAGG GGAGCAAACGGG	Megasphaera	Responders	2	0.143
TACGGAGGATGCGAGCGTTATCCGGATTTATTGGGTTTAAAGGGTGCGT AGGTTGTTTTTTAAGTCAGCGGTGAAAGTTTGTGGCTTAACCATAAAATT GCCGTTGAAACTGGGAGACTTGAGTGTGTTTGAGGTAGGCGGAATGTG TGGTGTAGCGGTGAAATGCATAGATATCACGCAGAACTCCAATTGCGA AGGCAGCTTACTAAACCATAACTGACACTGAAGCACGAAAGCGTGGGG ATCAAACAGG	Parabacteroides	Responders	2	0.138
TACGGAGGATGCGAGCGTTATCCGGATTTATTGGGTTTAAAGGGTGCGTAGGCGGC ACGCCAAGTCAGCGGTGAAATTTCCGGGCTCAACCCGGACTGTGCCGTTGAAACTG GCGAGCTAGAGTGCACAAGAGGCAGGCGGAATGCGTGGTGTAGCGGTGAAATGCAT AGATATCACGCAGAACCCCGATTGCGAAGGCAGCCTGCTAGGGTGCGACAGACGCT GAGGCACGAAAGCGTGGGTATCGAACAGG	Barnesiella	Responders	2	0.043
TACGTAGGTGGCAAGCGTTGTCCGGATTTACTGGGTGTAAAGGGCGTGTAGCCGGG TCGGCAAGTCAGATGTGAAATCCACGGGCTTAACTCGTGAACTGCATTTGAAACTGT TGATCTTGAGTATCGGAGAGGCAATCGGAATTCCTAGTGTAGCGGTGAAATGCGTA GATATTAGGAGGAACACCAGTGGCGAAGGCGGATTGCTGGACGACAACTGACGGTG AGGCGCGAAAGCGTGGGGAGCAAACAGG	Ruminococcaceae	Responders	2	0.009

To examine if incorporating predicted metabolic functions of the gut microbiota could improve our classification approach, we utilized PICRUSt2,^34^ a methodology that allows for predicting gene or pathway abundances based on 16S data. Predicted pathway abundances did not perform better compared to 16S data in classifying responders from non-responders for all three supplements (Fig. S5C). Of note, while an optimally tuned model for Inulin had comparable classification accuracy compared to 16S data, the model is likely not meaningful due to overfitting (i.e., component 1 of the model utilizes 305 pathways out of the included 394 pathways). As both 16S and predicted functional pathway abundance data indicate that it may be possible to predict responders to Inulin, we then looked at whether responders to Inulin had a different predicted baseline pathway abundances to non-responders. To do this, we performed differential abundance analysis, however, we did not find any significant pathways (after adjustment for multiple comparison) that was different between responders and non-responders (top 20 most significant pathways shown in Table S3).

Our results indicate that predicting an individual’s response to specific dietary fiber based on SCFA quantification is not straightforward and potentially not feasible for all fiber types. Nevertheless, we successfully developed a model that could predict an individual’s responsiveness to Inulin with some degree of certainty.

### Fibre supplements differentially alter PBMC population and immune cell functional output

As dietary fiber and SCFA modulate host immunity, we examined how short-term dietary fiber supplementation affects the immune system in healthy individuals, and whether these effects are explicitly linked to an increase in SCFA. To do this, we took a subset of participants (*n* = 12) with increased stool SCFA post-supplementation and examined their blood T cells, B cells, monocytes, macrophages, dendritic cells, and natural killer cells using flow cytometry (gating strategy presented in Fig. S6). To determine whether each supplement influenced these immune parameters, we compared PBMC pre- and post-fiber supplementation for each supplement (where pre-supplement refers to PBMC samples collected immediately prior to intervention, i.e., at baseline or following washout).

**Figure 6.**
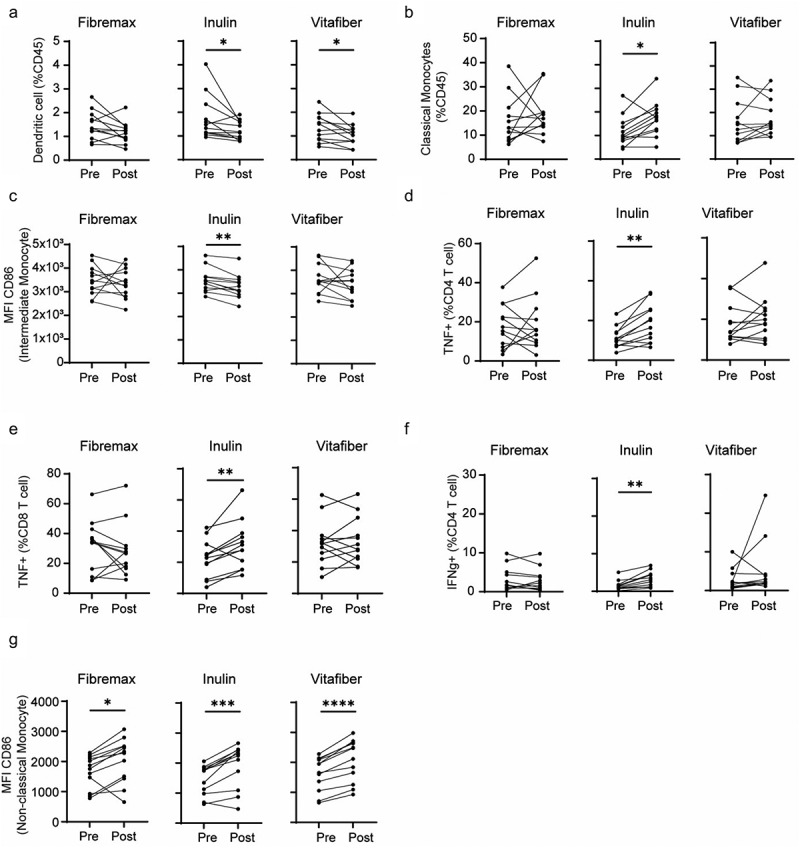
PBMC from *n* = 12 responder individuals (characterized by an increased in stool SCFA levels following fiber supplement) were immunophenotyped by flow cytometry. Comparison of PBMC between pre-supplementation (immediately prior to supplement intervention) vs. post-supplementation was made for each fiber supplement. (a-g) before-after plot of (a) dendritic cell, (b) classical monocytes, (c) CD86 expression (MFI) on intermediate monocytes, (d) CD86 expression (MFI) on non-classical monocytes, (e) TNF-producing CD4^+^ and (f) CD8^+^ T cells and (g) IFNγ-producing CD4^+^ T cells. *p <.05, **p <.01, ***p <.001 and ****p <.0001 as determined by paired t test.

We found that each fiber supplement had a unique effect on immune cells pre- vs. post supplementation (Table 5), indicating a differential effect of fiber supplements on immune cell activity. Consistent with its impact on the gut microbiota, Inulin had the strongest effect on the immune system, with a decreased proportion of circulating dendritic cells (Figure 6a) and an increased proportion of classical monocytes (Figure 6b). Inulin supplementation also affected the activation profile of immune cells with decreased expression of CD86, a costimulatory molecule, on CD16^hi^CD14^+^ intermediate monocytes (Figure 6c) and on immune cell cytokine production, with inulin supplementation leading to increased production of TNF in both CD4^+^ and CD8^+^ cells (Figure 6d–e), and IFNγ in CD4 T cells (Figure 6f). Some effects were not fiber specific, with all supplements leading to higher CD86 expression on non-classical monocytes (Figure 6g). Thus, fiber supplementation can differentially affect both the proportion and function of blood immune cells, with Inulin having the greatest impact.

**Table 5.** Immune parameters in PBMC of participant at baseline and following intervention with Fibremax, Inulin or Vitafiber supplement. Data represented as median (IQR). P-value represents paired t-test between baseline and intervention.

	Fibremax				Inulin				Vitafiber			
	Baseline	Intervention	Change	p-value	Baseline	Intervention	Change	p-value	Baseline	Intervention	Change	p-value
CD3+ T cells	48.05 (29.0, 54.8)	41.35 (33.28, 57.05)	−6.55 (−9.88, 6.68)	.224	47.25 (36.53, 60.05)	39.45 (32.2, 59.08)	−6.75 (−12.85, 2.28)	.084	46.6 (32.22, 56)	38.4 (34.18, 49.1)	−3.3 (−9.45, 3.78)	.456
CD4+ T cell (% of CD3+ T cell)	42.15 (29.5, 48.4)	43.5 (36.3, 50.9)	2.45 (−5.9, 5)	.307	47.4 (45, 48.5)	48.4 (46.5, 50)	0.7 (−1.4, 3.1)	.265	46.7 (35.2, 51.4)	44.9 (40.8, 50.8)	1.9 (−6.1, 6.60)	.725
CD4+ T cell	18.25 (14.03, 26.75)	20.15 (13.38, 24.93)	−1 (−6.2, 4.83)	.849	21.3 (16.8, 27.3)	16.65 (14.35, 28.23)	−2.1 (−7.84, 1.4)	.079	20.35 (15.58, 23.4)	17.55 (14, 23.85)	−1.65 (−5.3, 4.85)	.631
Regulatory T cell (% of CD4+ T cell)	2.81 (1.48, 3.52)	2.71 (1.55, 3.84)	−0.16 (−0.95, 0.76)	.707	2.83 (1.82, 3.99)	2.78 (1.89, 3.73)	−0.19 (−0.63, 0.23)	.139	2.28 (1.11, 3.97)	2.59 (1.54, 3.77)	0.19 (−1.16, 1.50)	.675
CD4+ Naïve T cell (% of CD4+ T cell)	45.55 (25.65, 75.48)	60.95 (45.78, 69.78)	−0.15 (−7.9, 16.33)	.377	56.3 (28.63, 67.53)	53.05 (39.15, 68.58)	0.4 (−2.7, 10.28)	.210	55.45 (31.75, 64.3)	56.2 (32.43, 65.1)	1.3 (−9.92, 6.98)	.954
CD8+ of T cell of total T cells	37.3 (29.48, 46.68)	34.25 (30.08, 45.53)	0.9 (−3.13, 3.48)	.478	35.2 (27.73, 44.05)	35.55 (30.88, 41.13)	0.40 (−1.3, 2.3)	.514	37.2 (30.35, 41.08)	37.45 (33.65, 43.7)	0.20 (−2.05, 4.58)	.524
CD8+ T cell	14.75 (12.55, 20.73)	16 (9.72, 18.85)	−1.41 (−3.08, 3.3)	.493	16.75 (12.03, 23.13)	14 (9.98, 19.18)	−2.03 (−2.9, 1.5)	.139	15.35 (11.58, 18.9)	14.05 (11.2, 16.78)	−0.27 (−2.1, 1.15)	.440
CD8+ Naïve T cell (% of CD8+ T cell)	57.55 (37.58, 80.33)	61.15 (36.45, 73.53)	−5.75 (−13.35, 0.99)	.695	64.75 (49.45, 69.4)	67.6 (53, 85.9)	−0.15 (−1.65, 18.63)	.280	69.35 (45.25, 83.78)	51.8 (33.73, 70.88)	−3.2 (−24.23, 2.63)	.090
B cell	14.8 (11.45, 18.23)	15.5 (10.95, 18.75)	−0.25 (−2.85, 1.76)	.972	13.65 (10.95, 16.98)	13.4 (10.68, 19.63)	0.11 (−2.58, 2.93)	.352	12.05 (10.09, 18.43)	13.05 (9.95, 16.85)	−0.050 (−0.738, 2.708)	.906
Memory B cell (% of B cell)	31.95 (24.18, 45.83)	25.05 (18.7, 40.58)	−3.05 (−6.58, 1.88)	.124	29.55 (22.45, 43.35)	27.25 (23.55, 40.53)	−0.55 (−3.93, 3.13)	.536	29.4 (22.55, 38.93)	30.55 (23.35, 44.43)	0.60 (−3.23, 3.68)	.495
Naive B cell (% of B cell)	53.9 (46.95, 64.68)	57.8 (50.95, 69.03)	1.9 (−1.33, 6.15)	.135	56.05 (48.13, 61.43)	59.85 (51.53, 62.28)	2.05 (−2.68, 4.88)	.341	58.75 (48.85, 64.05)	56.8 (48.6, 62.4)	−1.2 (−4.25, 3.5)	.622
Plasmablast (% of B cell)	1.5 (0.75, 2.37)	1.24 (0.90, 2.33)	0 (−0.53, 0.53)	.566	1.28 (0.98, 2.37)	0.98 (0.62, 1.48)	−0.41 (−0.78, 0.16)	.067	1.41 (0.66, 2.24)	1.15 (0.88, 1.51)	−0.095 (−0.605, 0.398)	.382
Transitional B cell (% of B cell)	3.93 (1.77, 8.18)	6.73 (2.78, 7.37)	0.72 (−0.01, 2.36)	.090	5.59 (2.57, 7.91)	5.13 (2.55, 8.49)	−0.33 (−1.15, 1.82)	.670	4.19 (1.92, 6.92)	3.91 (1.91, 7.53)	0.26 (−2.19, 0.70)	.366
Dendritic cell	1.34 (0.99, 1.87)	1.18 (0.87, 1.38)	−0.34 (−0.65, 0.03)	.074	1.41 (1.13, 2.18)	1.16 (0.89, 1.60)	−0.18 (−0.88, −0.15)	.043	1.37 (0.86, 1.78)	1.10 (0.78, 1.31)	−0.12 (−0.70, 0.04)	.024
Classical monocytes	13.1 (8.26, 20.52)	15.85 (13.73, 19.38)	2 (−4.41, 11.54)	.529	9.76 (7.96, 14.95)	17.1 (12.18, 20.73)	6.35 (1.05, 8.9)	.014	13.8 (7.78, 22.3)	14.8 (11.7, 24.4)	1.43 (−2.63, 5.76)	.399
Intermediate monocytes	5.48 (3.15, 6.70)	4.54 (3.25, 5.62)	−0.3 (−2.23, 0.64)	.210	4.33 (3.62, 7.71)	4.38 (3.218, 6.98)	−0.32 (−2.31, 0.59)	.382	4.87 (3.11, 7.31)	5.5 (3.87, 5.97)	0.32 (−1.18, 2.02)	.570
Non-classical monocytes	10.75 (7.23, 15.08)	11.65 (8.17, 13.98)	−1 (−2.33, 3.96)	.766	12.7 (7.95, 17.55)	13.35 (6.71, 16.8)	−1.36 (−2.4, 1.13)	.615	12.15 (9.15, 17.18)	12.9 (11.13, 15.45)	1.18 (−1.35, 3.38)	.310
CD86 expression (MFI): Dendritic cell	12074 (10829, 13449)	11377 (10210, 12702)	−533 (−2094, 686)	.301	11398 (10527, 13063)	10898 (10279, 12513)	−734 (−1542, 53)	.770	12705 (10720, 13866)	11705 (10390, 14170)	−383 (−1419, 1544)	.791
CD86 expression (MFI): Classical monocytes	22952 (18959, 28012)	22289 (18077, 23925)	−1383 (−3650, 1256)	.643	23416 (19920, 25874)	22281 (18623, 26376)	−1403 (−3321, 973)	.137	25074 (22164, 27484)	24733 (19628, 27461)	−426 (−3696, 798)	.750
CD86 expression (MFI): Intermediate monocytes	34269 (29971, 39262)	33371 (28482, 39551)	−2683 (−5228, 2988)	.688	35154 (31437, 37031)	32119 (29728, 35567)	−2126 (−4395, −1182)	.002	35409 (31055, 41742)	34078 (27817, 39310)	−2601 (−4934, 529)	.291
CD86 expression (MFI): Non-classical monocytes	1813 (1064, 2136)	2304 (1454, 2514)	475 (229, 655)	.0104	1755 (1018, 1821)	2280 (1250, 2429)	519 (260, 604)	.0004	1802 (1132, 2103)	2294 (1354, 2655)	448 (278, 546)	<.0001
Natural killer cell	8.87 (7.52, 13.75)	10.22 (7.69, 11.93)	−0.41 (−2.07, 3.19)	.743	11.2 (7.67, 15.9)	11.55 (5.09, 15.8)	−1.32 (−2.83, 2.09)	.694	12 (7.89, 15.88)	12.8 (9.30, 13.65)	0.995 (−1.1, 1.703)	.406
IFN-g+ CD4 T cell	1.69 (1.03, 4.90)	2.57 (0.96, 6.21)	−0.34 (−1.29, 1.66)	.378	1.16 (0.85, 1.74)	2.81 (1.18, 4.49)	1.22 (0.53, 2.67)	.0017	2.06 (0.97, 3.87)	2.19 (1.72, 4.11)	0.82 (−0.33, 1.39)	.267
IL-10+ CD4 T cell	0.69 (0.40, 1.70)	0.91 (0.47, 1.32)	0.035 (−0.675, 0.515)	.365	1.38 (0.28, 2.32)	0.67 (0.38, 1.23)	−0.12 (−1.90, 0.52)	.150	0.91 (0.51, 1.53)	0.84 (0.44, 1.22)	0.035 (−1.005, 0.193)	.216
TNF+ CD4 T cell	16.35 (9.55, 27.58)	15.95 (9.69, 25.33)	−1.09 (−9.53, 9.96)	.703	10.4 (7.22, 13.98)	18.25 (9.27, 25.5)	7.01 (4.44, 10.83)	.0012	13.7 (11.43, 23.83)	18.9 (11.6, 25.1)	0.449 (−2.353, 11.055)	.251
IFN-g+ CD8 T cell	17 (5.69, 24.5)	11.63 (7.86, 21.33)	−2.05 (−7.93, 0.52)	.255	12.85 (5.75, 15.48)	13.15 (8.86, 21.23)	3.34 (0.18, 7.3)	.0792	16.8 (12.33, 20.2)	16.15 (11.2, 21.25)	0.50 (−5.56, 5.15)	.909
IL-10+ CD8 T cell	0.315 (0.25, 0.56)	0.65 (0.26, 1.11)	0.29 (−0.038, 0.748)	.812	0.49 (0.22, 4.67)	0.315 (0.19, 0.67)	0.02 (−4.38, 0.30)	.127	0.46 (0.21, 0.73)	0.37 (0.22, 0.56)	−0.015 (−0.23, 0.08)	.208
TNF+ CD8 T cell	34.4 (12.25, 41.45)	27 (17.03, 31.33)	−3.20 (−10.13, 5.65)	.462	23.6 (11.32, 29.95)	29.55 (16.73, 38.05)	8.1 (5.15, 12.23)	.0055	31.9 (22.88, 36.28)	29.6 (22.45, 45.35)	2.9 (−8.95, 7.6)	.672
IFN-g+ B cell	0.34 (0.098, 2.033)	0.26 (0.17, 0.57)	−0.119 (−1.945, 0.203)	.241	0.41 (0.32, 2.12)	0.18 (0.12, 0.45)	−0.197 (−1.748, −0.008)	.178	0.49 (0.20, 1.32)	0.275 (0.22, 0.56)	−0.084 (−0.868, 0.081)	.221
IL-6+ B cell	0.355 (0.23, 0.84)	0.49 (0.28, 0.81)	0.18 (−0.64, 0.51)	.248	0.87 (0.46, 4.15)	0.45 (0.183, 0.99)	−0.575 (−3.608, −0.193)	.168	0.33 (0.18, 0.86)	0.475 (0.185, 1.393)	−0.13 (−0.70, 1.25)	.206
IL-10+ of B cell	1.19 (0.61, 2.0)	0.76 (0.38, 1.53)	−0.36 (−1.07, 0.11)	.082	0.95 (0.71, 2.11)	0.63 (0.47, 1.6)	−0.34 (−0.86, −0.14)	.160	1.15 (0.70, 1.68)	0.78 (0.51, 1.41)	−0.21 (−1, 0.24)	.266
TNF+ of B cell	40.2 (23.5, 54.68)	35.15 (24.03, 53.05)	−9.35 (−14.35, −0.1)	.848	32.05 (22.93, 39.73)	39.65 (21.73, 58.9)	11.65 (−10.9, 19.9)	.290	42.1 (24.13, 49.33)	45.45 (23.43, 52.93)	−1.75 (−15.55, 10.65)	.924

## Discussion

We show that one week of fiber supplementation in healthy women is sufficient to induce specific changes both at the levels of the gut microbiota and of the immune system. These responses were individualized and depended on the type of dietary fiber used. The product Inulin (fructo-oligosaccharide) elicited the most consistent and predictable effect across individuals, whereas Vitafiber had the least effect. Using a machine learning approach, we were able to identify taxa that were associated with SCFA production in response (*Alistipes marseilloanorexicus*) or not (*Senegalimassilia anaerobia* and *Oscillibacter spp*.) to supplementation with Inulin. Future large-scale studies are required to confirm these findings and to confirm that these taxa could be used to select the most appropriate dietary fiber for an individual to maximize SCFA production. Resolving the gut microbiota at the species level may further improve this approach, as 16S sequencing does not provide adequate species and strain level resolution.^35^

Dysbiosis is associated with many diseases. Dietary fiber can restore gut microbiota composition and function, and therefore holds promise as a tool to prevent or alleviate diseases. However, human clinical trials using dietary fiber have shown varying success in recapitulating the beneficial effects of fiber demonstrated in preclinical studies.^19–21^ This is likely explained by the high inter-individual heterogenicity of the human gut microbiota, each possessing a unique repertoire of enzymes with varying abilities to break down different fiber. Indeed, the ability to generate SCFA from specific fiber is dependent on an individual’s microbiome^36^ and improvement in glucose metabolism following fiber intake was only shown in individuals with increased abundance of *Prevotella*.^29^ However, a recent study reported no correlation between microbial metagenomic gene abundance and microbiota-derived metabolite levels.^37^ This suggests that it may not be possible to create a reliable model for predicting SCFA production based on either gut microbiota composition or its functional profile. Other strategies, such as examining the presence of interactions between different bacteria, may be required.

Despite its overall stability, our study showed that aspects of the human gut microbiome are modifiable. One week of inulin supplementation was sufficient to promote an increase in the relative abundance of specific bacterial taxa, particularly *Actinobacteria*, mostly due to increases in *Bifidobacterium* species. These are widely considered potent probiotics and high SCFA producers.^38^ This is consistent with the notion that dietary fiber affects the gut microbiota within days.^39^ Furthermore, known SCFA producers were signature to post-supplement microbiome, including *Anaerostipes hadrus*^40^ for Inulin, and taxa belonging to the genera Blautia^41^ and Clostridium cluster IV^42^ for Fibremax. These characteristics were lost following a 2-week washout period, suggesting that this timeframe was sufficient to revert diet-induced microbiome changes in healthy adults. This is consistent with previous studies, whereby inulin supplementation increased *Bifidobacteria* that peaked within a week and progressively normalized within a one-to-two-week period once supplementation stops.^43^ Our results indicate that the consumption of prebiotics must be continuously maintained for their beneficial effects on the gut microbiota composition to persist.

Gut microbiota diversity was most reduced by Inulin supplementation, followed by Fibremax, whereas Vitafiber had no effect. High-fiber diet has been shown to reduce microbiota diversity in mice^5^ and in humans.^44^ This may be explained by the selection of bacteria that thrive on the source of energy substrates provided by a particular diet.^45^ In the present study, participants were asked to maintain their usual diet, suggesting that fiber supplementation was sufficient to recapitulate the effects of a high-fiber diet on gut microbiota diversity, at least with Inulin. These results are contradictory to those of other studies, which reported an increase in gut microbiome diversity with high fiber intake, including in mice^24^ and humans.^25^ A recent study reported that microbiota diversity did not increase with a high-fiber diet, but rather with a diet high in fermented foods^26^. One differentiating factor between studies may be the dose and type of fiber used. Very high fiber intake creates an environment that selects for fiber-degrading bacteria, allowing them to outcompete other bacteria^46^ and reduce the overall microbiome diversity. As microbial diversity is often quoted as beneficial, our study indicates that this may be context-dependent, and reduced diversity is not necessarily an indicator of poor health.^44^ For example, a high-fiber diet intervention was shown to improve glucose homeostasis in type 2 diabetes patients despite a decreased richness of the gut microbiota.^44^

A more important readout may be the functional profile of the gut microbiome and its ability to degrade fiber that provide maximal SCFA for the host. Our study found that all three supplements had differential impact on PBMCs. SCFA typically have anti-inflammatory effects, including induction of tolerogenic dendritic cells and immunosuppressive regulatory T cells.^5,47,48^ We did not observe changes in regulatory T cells in PBMC but instead found that Inulin increased the proinflammatory potential of T cells, including increased TNF and IFNγ production. Regulatory T cells have been reported to increase with fiber and SCFA; however, these were in the gastrointestinal tract.^5,48,49^ While dietary fiber is typically associated with anti-inflammatory effects, inulin has been shown to drive type 2 inflammation in a mouse model of allergic airway inflammation, mediated by gut microbial production of secondary bile acids.^50^ In contrast, our observed pro-Th1 effect of inulin under healthy conditions is consistent with the finding that inulin could prevent allergy development in mice.^51^ Inulin may have differential effects in mice and humans or its impact on the immune system depends on the challenge encountered. Indeed, our data suggests that the beneficial effects of short-term dietary fiber supplementation on immunity might be context dependent. For example, Inulin could be beneficial in cancer as previously reported based on its impact on IFNγ and TNF response.^52,53^ However, these cytokines are known to aggravate autoimmune diseases, highlighting the importance of personalized interventions to avoid disease aggravation.

Differences in responses to fiber may also be explained by the status of the host. In our study, Vitafiber had the least observable impact on the gut microbiota composition, diversity, SCFA production, and immune phenotypes. This is likely because Vitafiber, composed of corn starch rich in soluble fiber, closely represents common dietary ingredients. Success with fiber supplementation, therefore, also relates to the individual’s dietary habits and microbiome profile. It also highlights a potential limitation of our study, which was performed in healthy participants and did not control for other aspects of diet. It also raises the question of whether individuals with dysbiosis would respond similarly to fiber supplements such as inulin. This aspect is particularly relevant as dysbiotic mice supplemented with inulin developed icteric hepatocellular carcinoma^54^ and fiber supplementation in patients with inflammatory bowel disease elicited a proinflammatory effect due to the inability of a dysbiotic microbiota to ferment the fiber, leading to increased intact β-fructan that activates NLRP3 and TLR2 pathways.^55^ These findings suggest that the health status of individuals and of their gut microbiota can switch the effects of supplements from beneficial to detrimental, which could raise concerns regarding the safety of these food products. Long-term deprivation of fiber, particularly across generations, can lead to irreversible loss of fiber digesting bacterial species.^24^ Therefore, alternative approaches, such as synbiotic intervention, or prior fecal microbiota transplant, may be required to rescue individuals with a dysbiotic gut microbiota. For example, while the treatment success of fecal microbiota transplant has only been reliably demonstrated for *Clostridium difficile* infections, the impact of fecal microbiota transplant on recipients’ gut microbiota composition appears to be long-lasting, with close resemblance to donor microbiota composition even after 6 months.^56^ As we have shown that gut microbiota composition can be predictive of a response to fiber supplement (at least with Inulin), this technique holds potential promise in restoring a healthy gut microbiome. Whether transplanting a fiber-responsive microbiota into individuals can improve their response to fiber and to promote higher SCFA production would need to be investigated in future studies.

By utilizing a cross-over design involving three different fiber supplements, we were able to show that fiber supplements have both universal and individualized effects across clinical, microbiota, and immune characteristics in healthy individuals. Our study indicates that different fiber supplementation should be personalized based on an individual’s gut microbiota composition and on the desired outcome.

## Methods

### Subjects and sampling

A total of 34 female volunteers aged between 30 and 65 years old, with a body mass index (BMI) ranging from 18 to 30 kg/m^2^ were recruited, and *n* = 28 completed the study. Exclusion criteria were type 1 or type 2 diabetes mellitus, renal or liver disease, cancer or active neoplasms, hyperthyroidism (unless treated or under control), use of medications known to affect weight or energy expenditure, unintentional weight loss (>10% body weight) over the past 5 years, smoking, alcohol consumption (>3 drinks/day), food allergies and/or intolerances, and when changes in diet are contraindicated by the treating doctor.

Volunteers were recruited through electronic and paper media advertisements, including social media and pamphlets, and assessed for eligibility through a screening questionnaire and a screening visit at the Clinical Research Facility at the Charles Perkins Centre. The screening questionnaire included questions regarding medical history, medications, supplements, allergies, and intolerances. Weight and height were measured according to standardized protocol, and BMI was calculated (weight/height)^2^. Rolling recruitment and assessment were conducted between February 2018 and April 2019.

### Ethics

The trial was conducted in accordance with the Declaration of Helsinki and all procedures were reviewed and approved (Protocol Number X17–0130 & HREC/17/RPAH/192) by the Ethics Review Committee (Royal Prince Alfred Hospital Zone) of the Sydney Local Health District. The trial was registered with the Australian and New Zealand Clinical Trials Registry (ACTRN12617001139369) on the 21^st^ of November 2016. All participants signed a consent form.

### Fibre supplements

Three different dietary fiber supplements were used for this study and were purchased commercially: Vitafiber (Isomalto-oligosaccharide; Myprotein), Inulin (fructo-oligosaccharide; Myprotein), and Fibremax (47% chicory root extract, 23.5% psyllium husk, 23.5% soy Fiber, 5% oat bran, and 1% Pectin; New Image). Participants were asked to consume the fiber supplements three times daily, a total of 15 g of Vitafiber, 34 g of Fibremax, or 15 g of inulin, equating to approximately 15 g of soluble fiber per day. During the intervention, participants were provided a 7-day dairy where they indicated the time(s) they took the supplement each day. They were also asked to record any adverse symptoms (bloating, gas and diarrhea) and their dietary intake. Dietary intake (habitual and during intervention) was determined through collection of a four-day estimated food record (4dEFR). Participants were required to record their dietary intake for four consecutive days (including one weekend day) giving as much detail about food consumed as possible. This included brands, preparation technique, leftovers (bones, skin, core), recipes and food consumed outside of home. Dietary intake data was analyzed using FoodWorks 10 Professional, v10.0. Brisbane: Xyris Pty Ltd, 2019 with the AUSNUT 2011‒13 database (Food Standards Australia New Zealand (2014). AUSNUT 2011–13 – Australian Food Composition Database. Canberra: FSANZ. Available at www.foodstandards.gov.au).

### Stool collection

Stool samples were collected within 48 hours prior to each clinical assessment. Participants were provided with a thermal bag (Thermabag), a small icepack, and written instructions on sample collection, storage, and transportation. Participants were asked to record the time of the collection and to place the sample in the freezer immediately after collection. Participants were requested to minimize the time out of the freezer to less than 2 hours when delivering to the clinic on the assessment day. Once received, the samples were stored in −80°C freezers.

### Blood collection and processing

Four-hour fasting blood samples were collected on the day of the clinical assessments. Blood samples were collected into 10 mL BD Vacutainer K2-EDTA collection tubes (Becton Dickinson, #367525) for plasma and 8.5 mL BD Vacutainer SSTII advance collection tubes (Becton Dickinson, #367958) for serum, centrifuged immediately (4500 rpm, 15 min at 4°C), pipetted into CryoPure cryogenic tubes (Sarstedt AG & Co, #72.380), and stored at − 80°C. For the isolation of peripheral blood mononuclear cell (PBMC) for immune phenotyping, the SepMate™ PBMC Isolation Tubes (StemCell Technologies, #85460) with Lymphoprep™ (StemCell Technologies, #07861) was used to isolate PBMC from whole blood, according to the manufacturer’s instructions. Purified PBMC were cryopreserved in 10% DMSO (Sigma-Aldrich, #276855) in fetal bovine serum (FBS) (Bovogen, #SFBS-AU).

### Metabolite quantification from stool and plasma

SCFA levels in plasma and stool were measured by nuclear magnetic resonance (NMR) analysis. Briefly, samples were filtered through a 3 kDa membrane filter (Merck Millipore, #UFC500396), and polar metabolites was extracted from the aqueous phase of a water:chloroform:methanol mixture (Sigma-Aldrich, #151858 and #151947) and diluted in trisodium phosphate (Sigma-Aldrich, #342483) buffer (pH 7). Samples were analyzed on a Bruker 600 MHz NMR machine containing 0.5 mM 4,4-dimethyl-4-silapentane-1-sulfonic acid (Sigma-Aldrich, #178837) as an internal standard. Data were analyzed using the Chenomx Profiler software (8.31). Stool samples were first homogenized in deuterium oxide at a final concentration of 100 mg/ml before filtration.

### Immune phenotyping of PBMC

Cryopreserved PBMC were thawed and washed with RPMI medium (Gibco, #21870092) containing 10% FBS, 2 mM L-glutamine (Gibco, #25030081), 100 units/mL penicillin, and 100 µg/mL streptomycin (Gibco, #15140122). 1 × 10^6^ cells were stained with Fc block (BioLegend, #422302) and the Live/Dead Fixable Blue Dead Cell Stain Kit (Invitrogen, #L34962) for 30 min at 4°C and washed with FACS buffer (2% FBS in PBS). For each participant, samples from each timepoint were first barcoded by staining with a combination of different fluorophore-conjugated CD45, washed, and then pooled for extracellular and intracellular staining. For staining of intracellular targets, PBMC were first stimulated with 50 ng/ml phorbol 12-myristate 13-acetate (Sigma-Aldrich, #P8139), 500 ng/ml ionomycin (Sigma-Aldrich, #I0634) and 5 µg/ml brefeldin A (Sigma-Aldrich, #B6542) for 4 h prior to staining. The Foxp3/Transcription Factor Staining Buffer Set (eBioscience, #00-5523-00) was used for intracellular cytokine staining. The following fluorescent-conjugated anti-human antibodies were used in this study: CD24-BUV395 (ML5), CD27-BUV661 (M-T271), CD45-BUV737 (HI30), CD38-BUV805 (HB7), CD19-V450 (HIB19), CD16-BV510 (3G8), CD86-PE/Cy5 (FUN-1), CD56-PE/Cy7 (B159), CD31-R718 (L133.1), CD3-APC-H7 (SK7) from BD Biosciences, CD45-BV570 (RA3-6B2), CD11c-BV605 (3.9), CD45-BV650 (HI30), CD45RA-BV711 (HI100), CD4-BV785 (OKT4), FoxP3-AF488 (259D), CD8-PerCP/Cy5.5 (SK1), CD14-APC (HCD14), IL-10-BV421 (JES3-9D7), TNF-PE (MAb11), IFNγ-PE/Cy7 (4S.B3) from BioLegend, CD25-PE (BC96) from Invitrogen and IL-6-APC (REA1037) from Miltenyi Biotec. Samples were acquired on a Cytek 5 L Aurora spectral flow cytometer, and data were analyzed using FlowJo v10.

### Gut microbiota analysis

DNA was extracted using the FastDNA™ SPIN Kit for Feces (MP Biomedicals #116570200), following the manufacturer’s instructions. Amplifiability and DNA concentration were verified by PCR and the Qubit assay kit (Invitrogen). Illumina sequencing of the V4 region (515f-806 r) of the 16S rRNA gene was performed commercially at the Ramaciotti Centre for Genomics (The University of New South Wales). Paired-end reads (2 × 250 bp) were processed with the dada2 package (1.12.1) using R software (version 3.6.1). Briefly, forward and reverse reads were trimmed (F240; R200) and quality-filtered (using default parameters) to remove low sequence bases, and error model was determined (10^8^ bases). Sequences were then dereplicated and exact amplicon sequence variants (ASVs) were inferred before merging of paired-end reads and removal of chimeric sequence. Taxonomy was assigned using the Ribosomal Database Project (RDP) training set (rdp_train_set_16) with specie-level taxonomy (dp_species_assignment_16) assignment (10.5281/zenodo.801828). The microbiome was explored using the following R packages phyloseq (1.42.0), microbiome (1.20.0), ALDEx2 (1.30.0), and mixOmics (6.22.0). Sequencing data were deposited in the European Nucleotide Archive under accession number PRJEB61931.

### Statistical analysis

To calculate the number of participants required for the study, we estimated a dropout rate of approximately 10% and a within-subject correlation of changes to stool acetate of 0.5 (SD between supplement group). A total of 30 subjects will be required to detect a difference with 80% power and a bilateral alpha risk of 5%.

For the analysis of clinical characteristics, fixed effects of treatment (supplement) and time (before versus after intervention) were performed using linear mixed-effects models, with each individual participant as a random effect. To compare pre- vs. post-supplement characteristics for an individual supplement, a two-tailed paired t-test was used. For the comparison of post-supplement characteristics between supplements, one-way ANOVA with Tukey’s multiple comparison test was used. To evaluate whether the overall microbiome composition differed between groups, a PERMANOVA test was used. For differential abundance analysis of microbiome composition between pre- and post-supplement microbiome, and between post-supplement microbiome between supplements, paired or unpaired ALDEx2 tests were used, respectively. Sparse partial least squares discriminant analysis (sPLSDA) was used for feature selection and classification, and the models were tuned using M-fold cross-validation with five folds for each validation and 100 repeats. The Balanced Error Rate was used as a misclassification measure and “max.dist” as the distance metric. Multilevel analysis (repeated measurement of participants across different interventions) was used, where appropriate. An alpha cutoff of 0.05 was used for all statistical tests. Data were analyzed using R Software (4.2.2) or GraphPad Prism 9 software.

## Supplementary Material

Supplemental Material
