# Supplementary material for: Functional profiling of gut microbial and immune responses toward different types of dietary fiber: a step toward personalized dietary interventions
Source: Gut Microbes. 2023 Nov 9;15(2):2274127. doi: 10.1080/19490976.2023.2274127 (PMC10730188; doi:10.1080/19490976.2023.2274127)
Supplement: Supplemental Material [file KGMI_A_2274127_SM0404.docx]

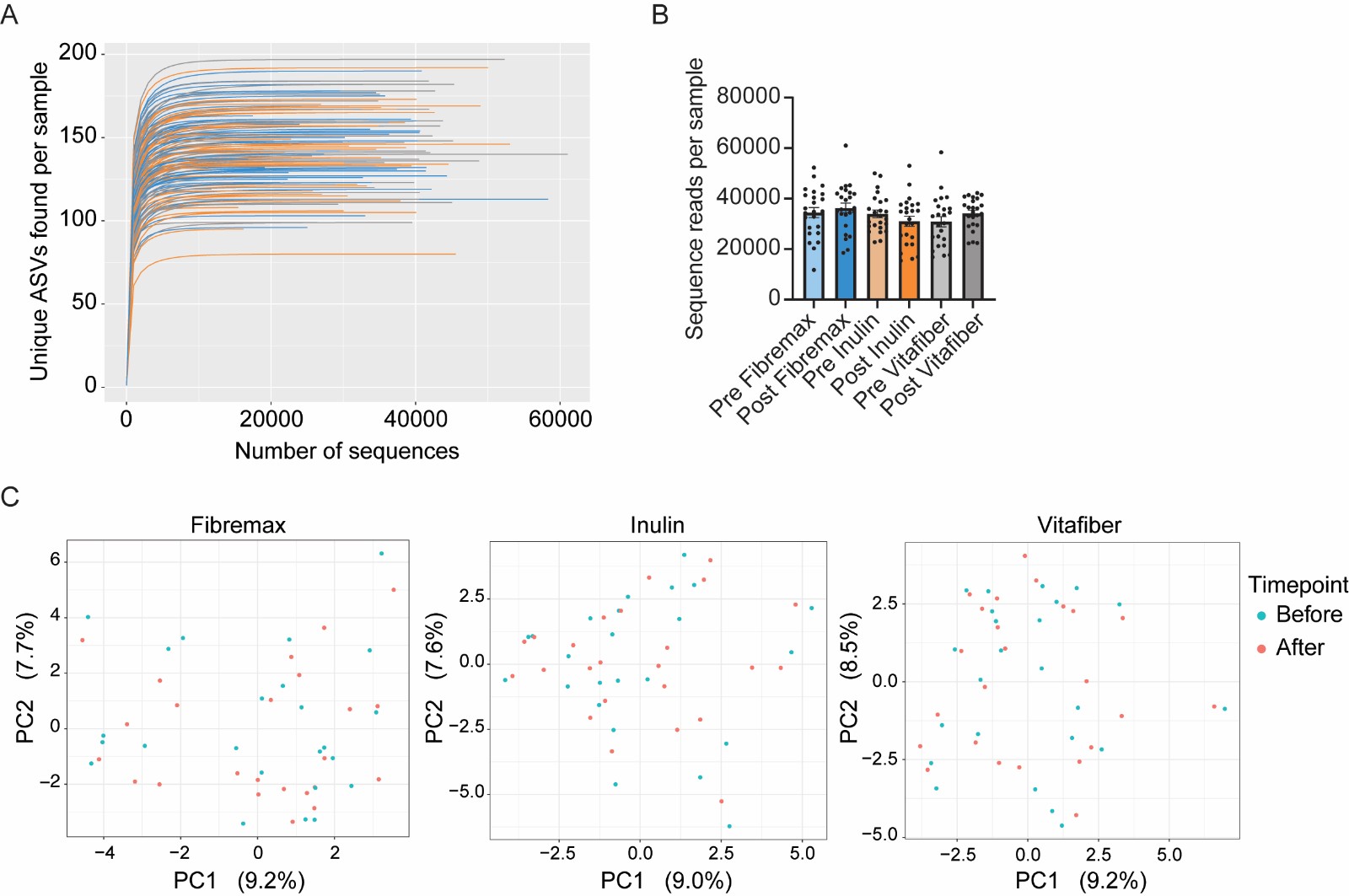


**Supplementary Figure 1:** Stool were collected before and one-week following consumption of either Fibremax, Inulin or Vitafiber and 16S rRNA gene sequencing was performed to analyze gut microbiota composition. **(A)** Rarefaction analysis which highlights the unique number of amplicon sequence variant (ASV) found per sample at indicated number of sequences (reads). **(B)** Total number of sequencing reads per sample. Data represented as mean +/- SEM **(C)** Differences in the overall microbiota communities of pre- vs. post-intervention Fibremax, Inulin and Vitafiber samples were analyzed by principal component analysis (PCA) of Aitchison’s distance. No significant clusters between pre- and post-intervention samples were observed, and as determined by PERMANOVA analysis (p>0.05).


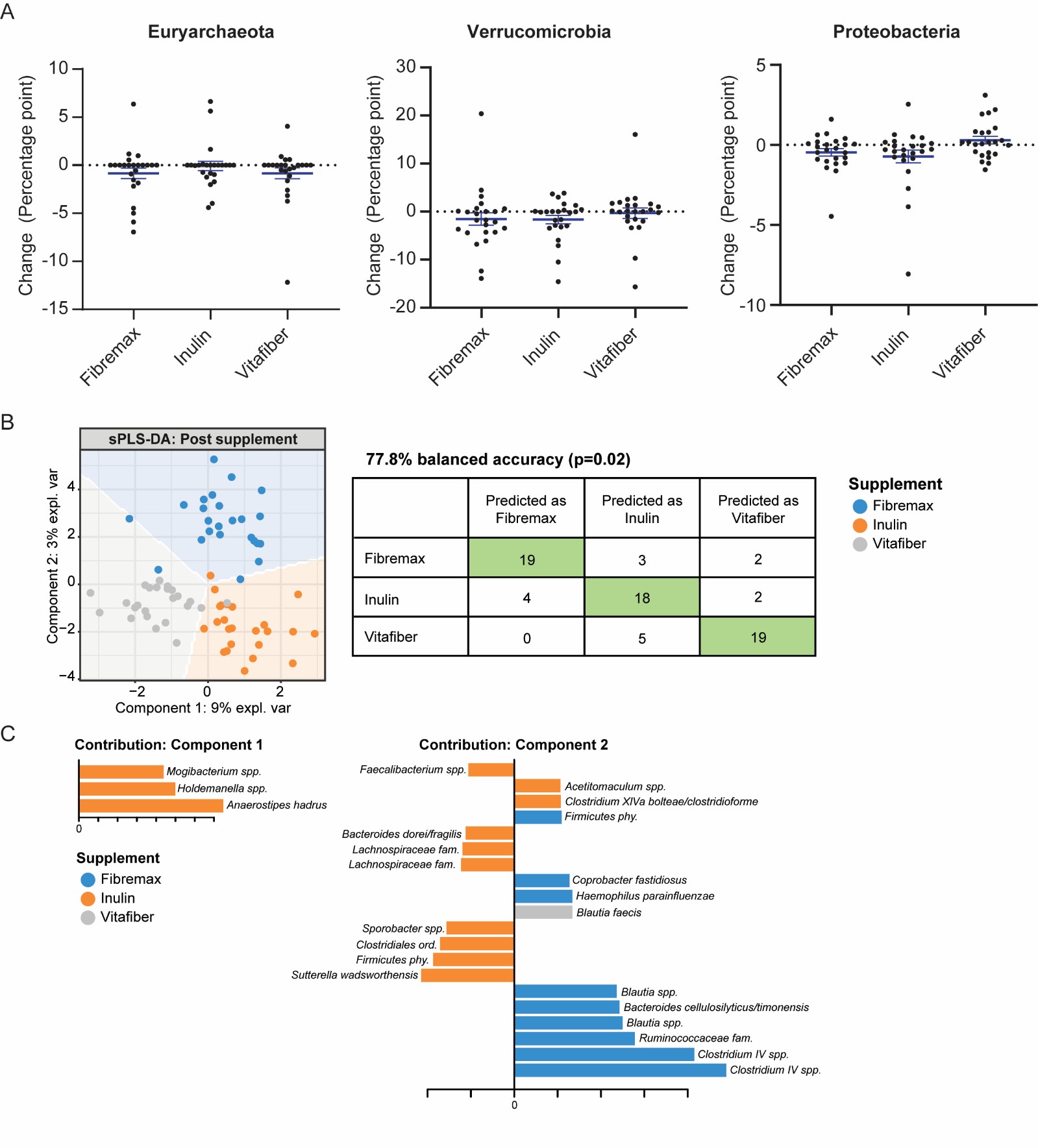


**Supplementary Figure 2:** Stool were collected before and one-week following consumption of either Fibremax, Inulin or Vitafiber and 16S rRNA gene sequencing was performed to analyze gut microbiota composition. The absolute percentage change of **(A)** *Euryarchaeota*, *Verrucomicrobia* and *Proteobacteria* following intervention with either Fibremax, Inulin or Vitafiber. Data represented as mean +/- SEM **(B)** Scatter plot of sPLS-DA analysis illustrating the predicted gut microbiome composition of post-supplement samples (left). Confusion matrix outlining the performance of the prediction model (right) and **(C)** loading plots indicating the ASV used for constructing the sPLS-DA model, with each color representing its contribution to the different post-supplement microbiome and the length of the bar plots representing the importance to the model for each components (positive loading represents that the ASV is positively associated with the indicated post-supplement microbiome, while negative loading represents a negative association).


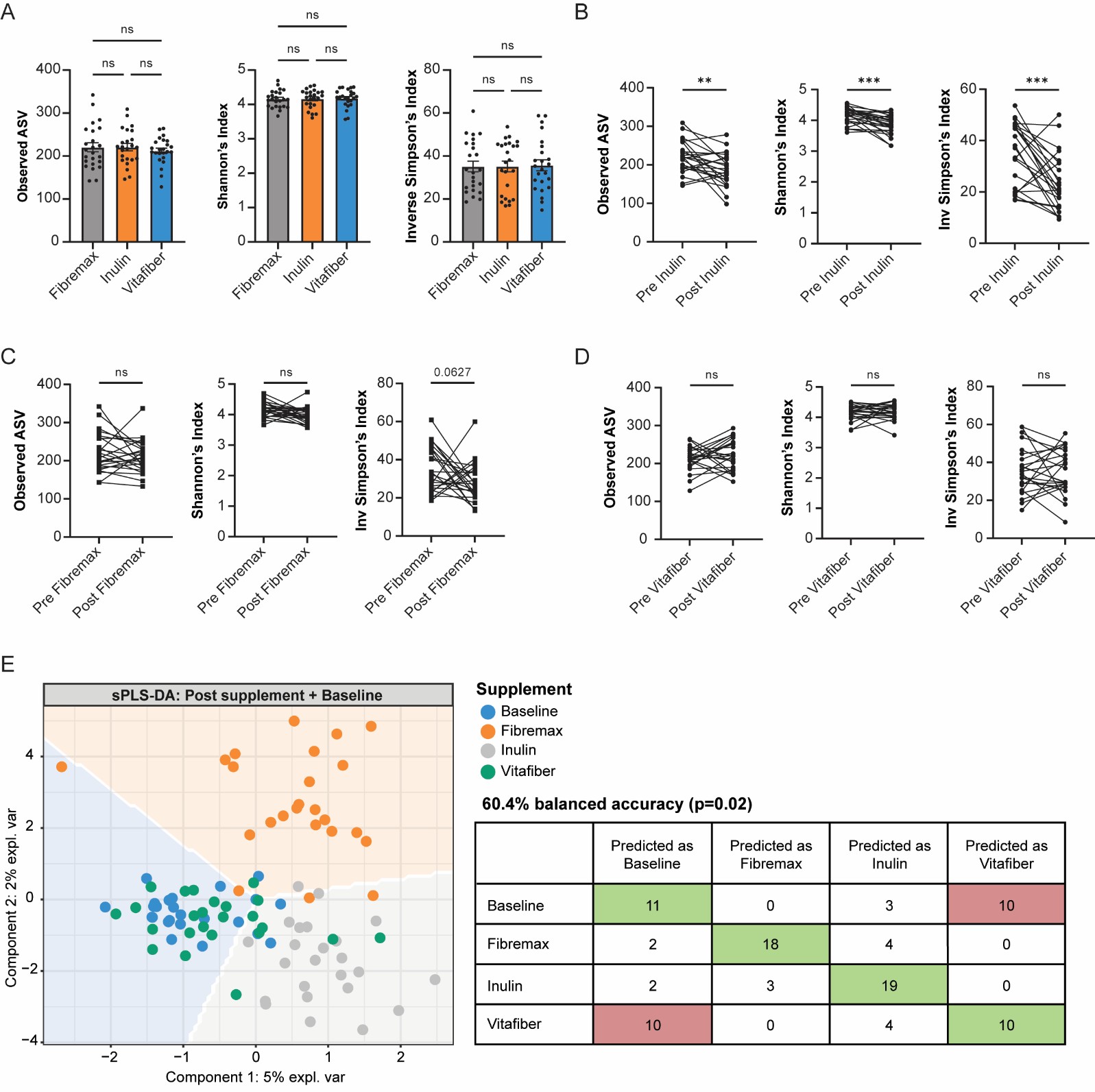


**Supplementary Figure 3:** Stool were collected before and one-week following consumption of either Fibremax, Inulin or Vitafiber and 16S rRNA gene sequencing was performed to analyze gut microbiota composition. **(A)** Alpha diversity measures of post-supplement gut microbiome as measured by richness (Observed ASV), Shannon’s diversity index and Inverse Simpson’s diversity index. **(B-D)** Change in diversity induced by **(B)** Inulin, **(C)** Fibremax and **(D)** Vitafiber as measured by richness (Observed ASV), Shannon’s diversity index and Inverse Simpson’s diversity index. **p<0.01 and ***p<0.001 as determined by paired t test. **(E)** PCA plot of sPLS-DA analysis illustrating the predicted gut microbiome composition of post- supplement and baseline samples (left). Confusion matrix outlining the performance of the prediction model (right).


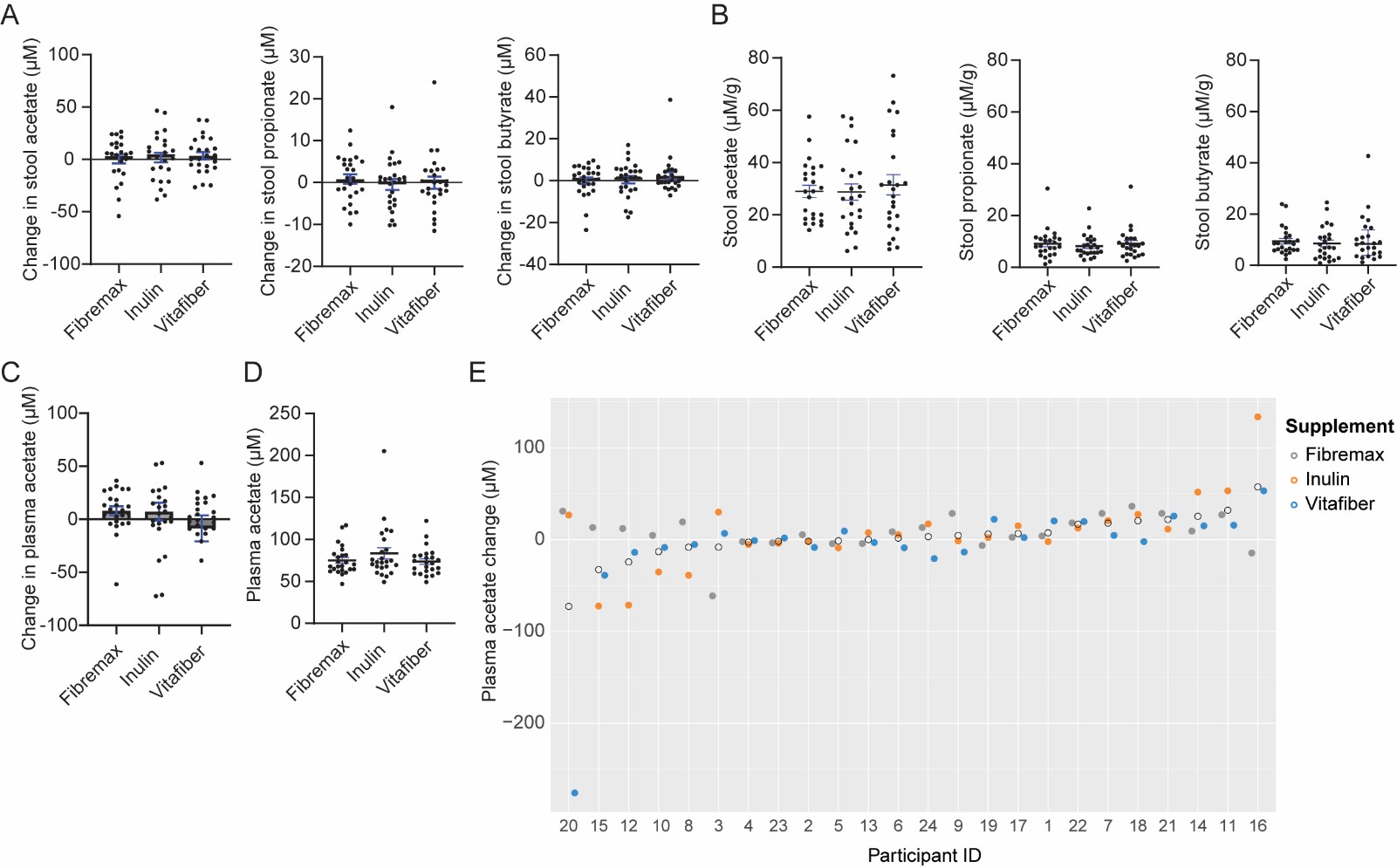


**Supplementary Figure 4:** Stool and plasma were collected before and one-week following consumption of either Fibremax, Inulin or Vitafiber and SCFA concentrations were quantified by nuclear magnetic resonance.

**(A)** The change in stool concentration of acetate, propionate and butyrate following one-week of Fibremax, Inulin or Vitafiber supplement were determined and **(B)** post-supplement stool concentration of acetate, propionate and butyrate was quantified. **(C)** The change in plasma concentration of acetate following one- week of Fibremax, Inulin or Vitafiber supplement were determined and **(D)** post-supplement plasma concentration of acetate was quantified. Data represented as mean +/- SEM. **(E)** Absolute change in plasma acetate for each participant is shown. Circles represent the mean change in plasma SCFA following intervention of each participant.


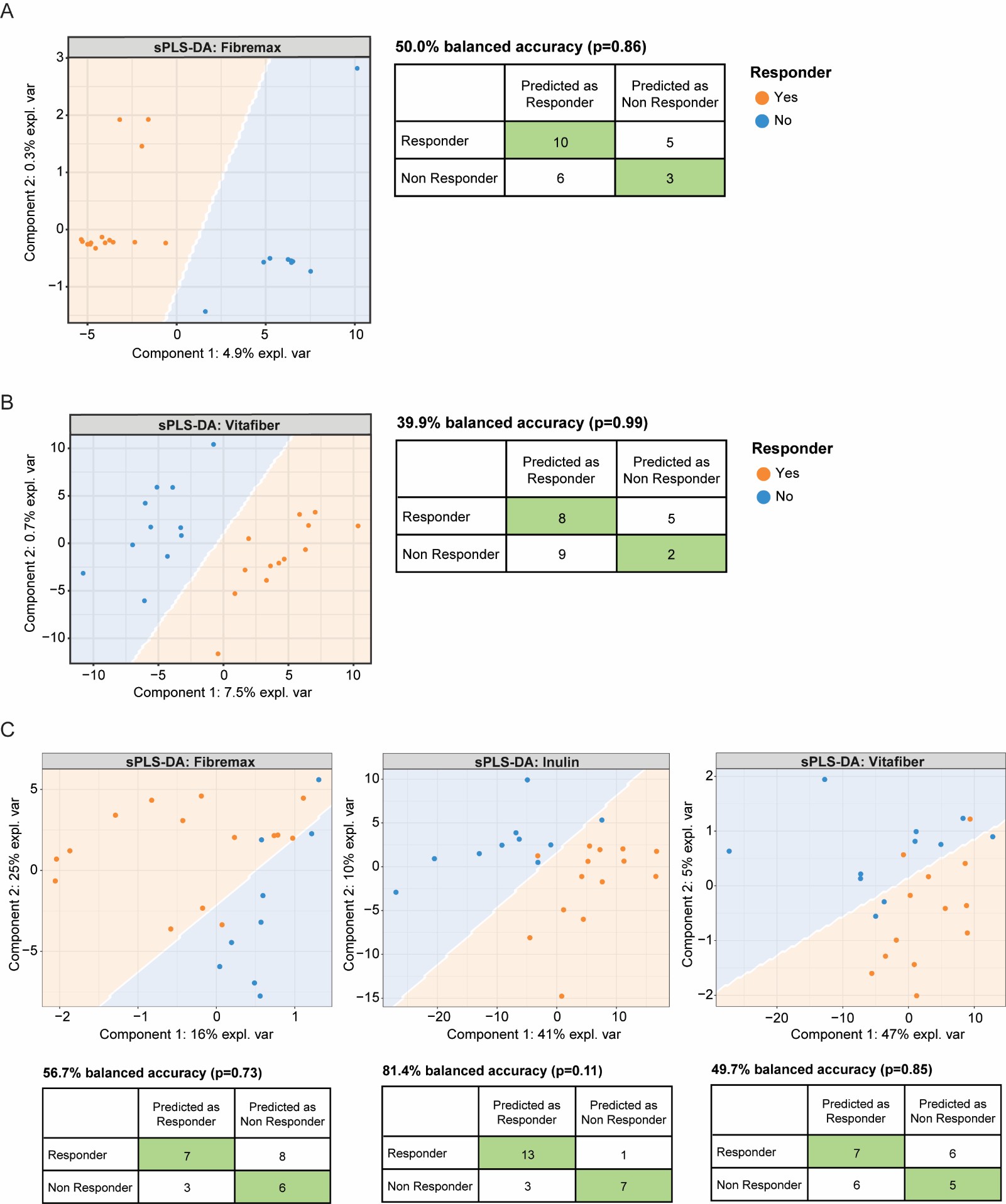


**Supplementary Figure 5: (A)** Scatter plot of sPLS-DA analysis illustrating the predicted gut microbiome composition of pre-supplement samples that were either responder or non-responders to Fibremax supplementation (left). Confusion matrix outlining the performance of the prediction model (right). **(B)** Scatter plot of sPLS-DA analysis illustrating the predicted gut microbiome composition of pre-supplement samples that were either responder or non-responders to Vitafiber supplementation (left). Confusion matrix outlining the performance of the prediction model (right). **(C)** Scatter plot of sPLS-DA analysis illustrating the predicted gut microbiota functional pathway extrapolated from 16S data using PICRUSt2 of pre-supplement samples that were either responder or non-responders to Fibremax, Inulin or Vitafiber supplementation (top) and confusion matrix outlining the performance of the prediction model (bottom).


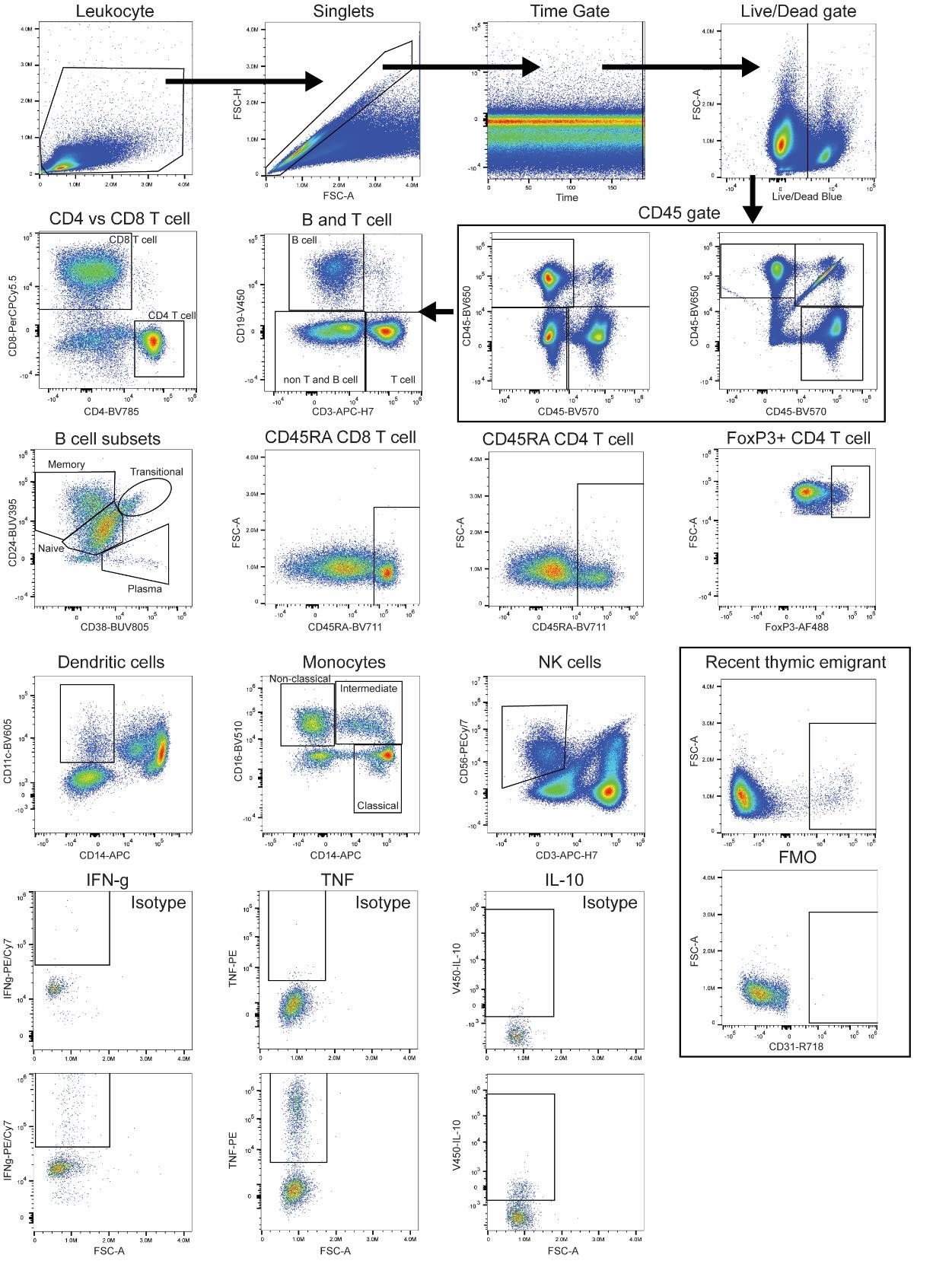


**Supplementary Figure 6:** Gating strategies used for the characterization of participant PBMC. For the identification of each immune subset, FSC-A and SCA-A was used to loosely gate on leukocytes and to exclude debris, followed by the gating of singlet cells (FSC-H vs. FSC-A), and a time gate was used to only include acquisition with consistent flow rate, then live cells were gated based on the Live/Dead blue staining. Finally, samples were gated on CD45 to identify leukocytes and to discriminate barcoded samples. Cells were then discriminated based on CD3 and CD19, and gating as either total T cells (CD3+CD19-), total B cells (CD3-CD19+) or non-T and B cells (CD3-CD19-). CD4 and CD8 T cells were identified as CD4+CD8- and CD4-CD8+, respectively, of total T cells and CD45RA was used to identify naïve CD4 and CD8 T cells, and CD31 used to identify recent CD4 or CD8 thymic emigrant T cells. For the identification of regulatory T cell, FoxP3 positive cells on CD4-gated T cells was used. NK cells were identified as CD3-CD56+ based on CD45+ gated population. For the identification of B cell subpopulations, CD24 and CD38 were used to characterize Naïve, Memory, Transitional as well as plasma B cells. For the identification of myeloid cells, population was gated based on the non-T and B cell gate: and dendritic cell characterized by CD14-CD11c+, monocytes as either classical monocytes (CD14++CD16-), intermediate monocytes (CD14++CD16+) or non- classical monocytes (CD14-CD16++). Expression levels of the cytokines IFN-gamma, TNF and IL-10 were gated based on corresponding isotype control antibody-stained sample.

**Supplementary table 1. Baseline and clinical dietary intake**

| **Dietary intake** | **Baseline (n=12)** | **Clinic 1 (n=24)** | **Clinic 2 (n=15)** | **Clinic 3 (n=16)** |
| --- | --- | --- | --- | --- |
| Energy, kJ (IQR) | 6783  (6325, 7468) | 6171  (5198, 7573) p=0.166 | 7287  (6275, 8414) p=0.838 | 6385  (4974, 8077) p=0.3444 |
| Protein, g (IQR) | 74.11  (63.59, 79.62) | 63.56  (51.5, 90.16) p=0.792 | 77.50  (48.48, 84.30) p=0.945 | 77.50  (59.84, 94.02) p=0.774 |
| Carb, g (IQR) | 172.1  (139.9, 188.8) | 155.0  (127.2, 195.0) p=0.350 | 187.6  (155.1, 207.3) p=0.749 | 159.9  (123.2, 185.0) p=0.231 |
| Fat, g (IQR) | 65.07  (58.0, 76.53) | 57.74  (45.78, 74.93) p=0.196 | 62.71  (56.15, 88.51) p=0.537 | 56.14  (45.72, 77.81) p=0.368 |
| Fibre, g (IQR) | 20.76  (16.17, 31.45) | 19.28  (16.16, 24.34) p=0.520 | 19.39  (12.95, 28.22) p=0.974 | 18.43  (16.03, 21.65) p=0.224 |

p-value represent student’s t-test in comparison to Baseline. Data reports participants who have completed their food diary at baseline and for each clinic.

**Supplementary table 2. Taxa (components) relating to sPLS-DA model of post-supplement microbiota.**

| **ASV** | **Taxa ASV belongs to** | **Contribute to group** | **Comp** | **Loading** |
| --- | --- | --- | --- | --- |
| TACGTAGGGGGCAAGCGTTATCCGGAATTACTGGGTGTAAAGGGTGCGTAG GTGGTATGGCAAGTCAGAAGTGAAAACCCAGGGCTTAACTCTGGGACTGCTT TTGAAACTGTCAGACTGGAGTGCAGGAGAGGTAAGCGGAATTCCTAGTGTAG CGGTGAAATGCGTAGATATTAGGAGGAACATCAGTGGCGAAGGCGGCTTACT GGACTGAAACTGACACTGAGGCACGAAAGCGTGGGGAGCAAACAGG | Anaerostipes hadrus | Inulin | 1 | 0.747 |
| TACGTAGGTGGCGAGCGTTATCCGGAATGATTGGGCGTAAAGGGTGCGTAG GTGGCAGATCAAGTCTGGAGTAAAAGGTATGGGCTCAACCCGTACTGGCTCT GGAAACTGATCAGCTAGAGAACAGAAGAGGACGGCGGAACTCCATGTGTAG CGGTAAAATGCGTAGATATATGGAAGAACACCGGTGGCGAAGGCGGCCGTC TGGTCTGGATTCTGACACTGAAGCACGAAAGCGTGGGGAGCAAATAGG | Holdemanella | Inulin | 1 | 0.499 |
| TACGTAGGGGGCAAGCGTTATCCGGAATTATTGGGCGTAAAGAGTGCGTAGG TGGTTATCTAAGCGTAAGGTTTAAGCGTGCAGCTCAACTGCATCTCGCCTTGC GAACTGGACTACTTGAGTGCAGGAGGGGAAAGCGGAATTCCTAGTGTAGCG GTGAAATGCGTAGATATTAGGAGGAACACCAGCGGCGAAGGCGGCTTTCTG GACTGTAACTGACACTGAGGCACGAAAGCGTGGGGAGCAAACAGG | Mogibacterium | Inulin | 1 | 0.439 |
| TACGTAGGGAGCGAGCGTTGTCCGGATTTACTGGGTGTAAAGGGTGCGTAG GCGGATAGGCAAGTCAGTGGTGAAATCTATGGGCTTAACCCATAAACTGCCA TTGAAACTGTTTATCTTGAGTGGAGTAGAGGCAGGCGGAATTCCCGGTGTAG CGGTGAAATGCGTAGAGATCGGGAGGAACACCAGTGGCGAAGGCGGCCTGC TGGGCTTTAACTGACGCTGAGGCACGAAAGTGTGGGTAGCAAACAGG | Clostridium_IV | Fibremax | 2 | 0.489 |
| TACGTAGGGAGCAAGCGTTGTCCGGATTTACTGGGTGTAAAGGGTGCGTAGG CGGTTTGGTAAGTCAGAAGTGAAATCCATGGGCTTAACCCATGAACTGCTTTT GAAACTATCGAACTTGAGTGAAGTAGAGGTAGGCGGAATTCCCGGTGTAGCG GTGAAATGCGTAGAGATCGGGAGGAACACCAGTGGCGAAGGCGGCCTACTG GGCTTTAACTGACGCTGAGGCACGAAAGCATGGGTAGCAAACAGG | Clostridium IV | Fibremax | 2 | 0.415 |
| TACGTAGGTGACAAGCGTTGTCCGGATTTACTGGGTGTAAAGGGCGCGTAGG CGGACTGTCAAGTCAGTCGTGAAATACCGGGGCTTAACCCCGGGGCTGCGA TTGAAACTGACAGCCTTGAGTATCGGAGAGGAAAGCGGAATTCCTAGTGTAG CGGTGAAATGCGTAGATATTAGGAGGAACACCAGTGGCGAAGGCGGCTTTCT GGACGACAACTGACGCTGAGGCGCGAAAGTGTGGGGAGCAAACAGG | Ruminococcaceae | Fibremax | 2 | 0.278 |
| TACGTAGGGGGCAAGCGTTATCCGGATTTACTGGGTGTAAAGGGAGCGTAGA CGGCGAGGCAAGTCTGATGTGAAAGCCTGGGGCTTAACCCCGGAACTGCAT TGGAAACTGCTTTGCTGGAGTGCCGGAGAGGTAAGCGGAATTCCTAGTGTAG CGGTGAAATGCGTAGATATTAGGAGGAACACCAGTGGCGAAGGCGGCTTACT GGACGGTAACTGACGTTGAGGCTCGAAAGCGTGGGGAGCAAACAGG | Blautia | Fibremax | 2 | 0.250 |
| TACGGAGGATCCGAGCGTTATCCGGATTTATTGGGTTTAAAGGGAGCGTAGG CGGACTATTAAGTCAGCTGTGAAAGTTTGCGGCTCAACCGTAAAATTGCAGTT GATACTGGTCGTCTTGAGTGCAGTAGAGGTAGGCGGAATTCGTGGTGTAGCG GTGAAATGCTTAGATATCACGAAGAACTCCGATTGCGAAGGCAGCTTACTGG ACTGTAACTGACGCTGATGCTCGAAAGTGTGGGTATCAAACAGG | Bacteroides cellulosilyticus/timo nensis | Fibremax | 2 | 0.243 |
| TACGTAGGGGGCGAGCGTTATCCGGATTTACTGGGTGTAAAGGGAGCGTAG ACGGCGTATCAAGTCTGATGTGAAAGGCAGGGGCTTAACCCCTGGACTGCAT TGGAAACTGGTATGCTTGAGTGCCGGAGGGGTAAGCGGAATTCCTAGTGTAG CGGTGAAATGCGTAGATATTAGGAGGAACACCAGTGGCGAAGGCGGCTTACT GGACGGTAACTGACGTTGAGGCTCGAAAGCGTGGGGAGCAAACAGG | Blautia | Fibremax | 2 | 0.236 |
| TACGTAGGGTGCAAGCGTTAATCGGAATTACTGGGCGTAAAGCGTGCGCAGG CGGTTCTGTAAGATAGATGTGAAATCCCCGGGCTCAACCTGGGAATTGCATA TATGACTGCAGGACTTGAGTTTGTCAGAGGAGGGTGGAATTCCACGTGTAGC AGTGAAATGCGTAGATATGTGGAAGAACACCGATGGCGAAGGCAGCCCTCTG GGACATGACTGACGCTCATGCACGAAAGCGTGGGGAGCAAACAGG | Sutterella wadsworthensis | Inulin | 2 | -0.215 |
| TACGTAGGGGGCAAGCGTTGTCCGGAATAATTGGGCGTAAAGGGCGCGTAG GCGGCTCGGTAAGTCTGGAGTGAAAGTCCTGCTTTTAAGGTGGGAATTGCTT TGGATACTGTCGGGCTTGAGTGCAGGAGAGGTTAGTGGAATTCCCAGTGTAG CGGTGAAATGCGTAGAGATTGGGAGGAACACCAGTGGCGAAGGCGACTAAC TGGACTGTAACTGACGCTGAGGCGCGAAAGTGTGGGGAGCAAACAGG | Firmicutes | Inulin | 2 | -0.187 |
| TACGTAGGGGGCGAGCGTTGTCCGGAATTACTGGGCGTAAAGGGAGCGTAG GCGGTCGATTAAGTTAGATGTGAAACCCCCGGGCTTAACTTGGGGACTGCAT  CTAATACTGGTTGACTTAGAGTACAGGAGAGGGAAGCGGAATTCCTAGTGTA | Clostridiales | Inulin | 2 | -0.171 |

| GCGGTGAAATGCGTAGATATTAGGAGGAACACCAGTGGCGAAGGCGGCTTT CTGGACTGACACTGACGCTGAGGCTCGAAAGCGTGGGGAGCAAACAGG |  |  |  |  |
| --- | --- | --- | --- | --- |
| TACGTAGGTGGCAAGCGTTGTCCGGATTTACTGGGTGTAAAGGGCGTGTAGG CGGAGCTGCAAGTCAGATGTGAAATCTCCGGGCTTAACCCGGAAACTGCATT TGAAACTGTAGTCCTTGAGTATCGGAGAGGCAAGCGGAATTCCTAGTGTAGC GGTGAAATGCGTAGATATTAGGAGGAACACCAGTGGCGAAGGCGGCTTGCT GGACGACAACTGACGCTGAGGCGCGAAAGCGTGGGGAGCAAACAGG | Sporobacter | Inulin | 2 | -0.156 |
| TACGTAGGGGGCAAGCGTTATCCGGATTTACTGGGTGTAAAGGGAGCGTAGA CGGCGCAGCAAGTCTGATGTGAAAGGCAGGGGCTTAACCCCTGGACTGCAT TGGAAACTGCTGTGCTTGAGTGCCGGAGGGGTAAGCGGAATTCCTAGTGTAG CGGTGAAATGCGTAGATATTAGGAGGAACACCAGTGGCGAAGGCGGCTTACT GGACGGTAACTGACGTTGAGGCTCGAAAGCGTGGGGAGCAAACAGG | Blautia faecis | Vitafiber | 2 | 0.134 |
| TACGGAGGGTGCGAGCGTTAATCGGAATAACTGGGCGTAAAGGGCACGCAG GCGGTGACTTAAGTGAGGTGTGAAAGCCCCGGGCTTAACCTGGGAATTGCAT TTCATACTGGGTCGCTAGAGTACTTTAGGGAGGGGTAGAATTCCACGTGTAG CGGTGAAATGCGTAGAGATGTGGAGGAATACCGAAGGCGAAGGCAGCCCCT TGGGAATGTACTGACGCTCATGTGCGAAAGCGTGGGGAGCAAACAGG | Haemophilus parainfluenzae | Fibremax | 2 | 0.134 |
| TACGGAAGATGCGAGCGTTATCCGGATTTATTGGGTTTAAAGGGTGCGTAGG CGGAAGAATAAGTCAGCGGTGAAATGCTTCAGCTCAACTGGAGAATTGCCGA TGAAACTGTTTTTCTAGAGTATAAAAGAGGTATGCGGAATGCGTGGTGTAGCG GTGAAATGCATAGATATCACGCAGAACCCCGATTGCGAAGGCAGCATACTGG GCTATAACTGACGCTGAAGCACGAAAGCGTGGGTATCGAACAGG | Coprobacter fastidiosus | Fibremax | 2 | 0.127 |
| TACGTATGGAGCAAGCGTTATCCGGATTTACTGGGTGTAAAGGGAGTGTAGG CGGCATGGCAAGTCTGATGTGAAAATCCGGGGCTTAACCCCGGACCTGCATT GGAAACTGTCAGGCTGGAGTGTCGGAGAGGTAAGTGGAATTCCTAGTGTAGC GGTGAAATGCGTAGATATTAGGAGGAACACCAGTGGCGAAGGCGGCTTACTG GACGACAACTGACGCTGAGGCTCGAAAGCGTGGGGAGCAAACAGG | Lachnospiraceae | Inulin | 2 | -0.123 |
| TACGTAGGGAGCGAGCGTTATCCGGATTTATTGGGTGTAAAGGGTGCGTAGA CGGGAATACAAGTTAGTTGTGAAATACCTCGGCTTAACTGAGGAACTGCAACT AAAACTATATTTCTTGAGTACAGGAGAGGTAAGTGGAATTCCTAGTGTAGCGG TGAAATGCGTAGATATTAGGAGGAACACCAGTGGCGAAGGCGACTTACTGGA CTGAAACTGACGTTGAGGCACGAAAGTGTGGGGAGCAAACAGG | Lachnospiraceae | Inulin | 2 | -0.119 |
| TACGGAGGATCCGAGCGTTATCCGGATTTATTGGGTTTAAAGGGAGCGTAGG TGGACTGGTAAGTCAGTTGTGAAAGTTTGCGGCTCAACCGTAAAATTGCAGTT GATACTGTCAGTCTTGAGTACAGTAGAGGTGGGCGGAATTCGTGGTGTAGCG GTGAAATGCTTAGATATCACGAAGAACTCCGATTGCGAAGGCAGCTCACTGG ACTGCAACTGACACTGATGCTCGAAAGTGTGGGTATCAAACAGG | Bacteroides dorei/fragilis | Inulin | 2 | -0.112 |
| TACGTAGGGGGCAAGCGTTGTCCGGAATGATTGGGCGTAAAGGGCGCGTAG GCGGCCAACTAAGTCTGGAGTGAAAGTCCTGCTTTTAAGGTGGGAATTGCTT TGGAAACTGGATGGCTTGAGTGCAGGAGAGGTAAGCGGAATTCCCGGTGTA GCGGTGAAATGCGTAGAGATCGGGAGGAACACCAGTGGCGAAGGCGGCTTA CTGGACTGTAACTGACGCTGAGGCGCGAAAGTGTGGGGAGCAAACAGG | Firmicutes | Fibremax | 2 | 0.109 |
| TACGTAGGGGGCAAGCGTTATCCGGATTTACTGGGTGTAAAGGGAGCGTAGA CGGCGAAGCAAGTCTGAAGTGAAAACCCAGGGCTCAACCCTGGGACTGCTTT GGAAACTGTTTTGCTAGAGTGTCGGAGAGGTAAGTGGAATTCCTAGTGTAGC GGTGAAATGCGTAGATATTAGGAGGAACACCAGTGGCGAAGGCGGCTTACTG GACGATAACTGACGTTGAGGCTCGAAAGCGTGGGGAGCAAACAGG | Clostridium XlVa bolteae/clostridiofor me | Inulin | 2 | 0.108 |
| TACGTAGGGGGCAAGCGTTATCCGGATTTACTGGGTGTAAAGGGAGCGCAG GCGGCAGGGCAAGTCAGATGTGAAAGCCCGGGGCTCAACCCCGGGACTGCA TTTGAAACTGTCCAGCTGGAGTACAGGAGAGGCAGGCGGAATTCCTAGTGTA GCGGTGAAATGCGTAGATATTAGGAGGAACACCAGTGGCGAAGGCGGCCTG CTGGACTGTAACTGACGCTGAGGCTCGAAAGCGTGGGGAGCAAACAGG | Acetitomaculum | Inulin | 2 | 0.107 |
| TACGTATGGAGCAAGCGTTATCCGGATTTACTGGGTGTAAAGGGAGTGTAGG TGGTATCACAAGTCAGAAGTGAAAGCCCGGGGCTCAACCCCGGGACTGCTTT TGAAACTGTGGAACTGGAGTGCAGGAGAGGTAAGTGGAATTCCTAGTGTAGC GGTGAAATGCGTAGATATTAGGAGGAACACCAGTGGCGAAGGCGGCTTACTG GACTGTAACTGACACTGAGGCTCGAAAGCGTGGGGAGCAAACAGG | Lachnospiraceae | Inulin | 2 | -0.106 |

**Supplementary table 3. Top 20 most differentially abundant PICRUSt2 predicted functional pathways in baseline microbiome between responders and non-responders to Inulin**

|  | **Median CLR**  **(Non-responders)** | **Median CLR (Responders)** | **Effect size** | **p value (Welch's t test)** | **Adjusted p value**  **(Welch's t test)** | **p value (Wilcoxon rank test)** | **Adjusted p value**  **(Wilcoxon rank test)** |
| --- | --- | --- | --- | --- | --- | --- | --- |
| **GLUCOSE1PMETAB-PWY** | -0.4168993 | 1.024869472 | 0.5852523 | 0.03720731 | 0.6886889 | 0.013107 | 0.5437466 |
| **RUMP-PWY** | 1.9134559 | 1.156825007 | -0.5926718 | 0.02532597 | 0.6863293 | 0.0125496 | 0.5437466 |
| **GLYCOL-GLYOXDEG-PWY** | -4.4524977 | -3.029141171 | 0.5148919 | 0.38299508 | 0.9061456 | 0.0231727 | 0.5441429 |
| **HEMESYN2-PWY** | 1.3649646 | 0.267172429 | -0.6591479 | 0.01618419 | 0.6863293 | 0.0217599 | 0.5454658 |
| **PWY-6876** | -1.7598251 | -3.284206702 | -0.6769241 | 0.01708571 | 0.6863293 | 0.0255148 | 0.5454834 |
| **PWY-5863** | -1.0265702 | 0.508438044 | 0.6808068 | 0.07449165 | 0.6911333 | 0.0320909 | 0.5463127 |
| **PWY-5861** | -0.1195632 | 1.008771749 | 0.6681835 | 0.06262748 | 0.6896838 | 0.0336828 | 0.5465456 |
| **PWY-5837** | -1.2165176 | 0.324318487 | 0.6601993 | 0.07529887 | 0.6905218 | 0.0322115 | 0.5468493 |
| **PWY-1861** | 3.08805 | 1.724251543 | -0.6111959 | 0.02574757 | 0.6863293 | 0.0304621 | 0.5472095 |
| **GLYOXYLATE-BYPASS** | -4.1746237 | -2.825749365 | 0.4853837 | 0.35897826 | 0.9088713 | 0.0287207 | 0.5475251 |
| **PWY-5840** | 0.3972896 | 1.327039269 | 0.5857726 | 0.05533224 | 0.6893671 | 0.0366452 | 0.5482247 |
| **PWY-5897** | 0.2342715 | 1.230510291 | 0.6576601 | 0.05615289 | 0.689187 | 0.0370279 | 0.5482904 |
| **PWY-5898** | 0.2231705 | 1.230045488 | 0.6083944 | 0.05535334 | 0.6893671 | 0.0366955 | 0.5483326 |
| **PWY-7392** | 2.7523307 | 1.784627803 | -0.5722562 | 0.06327296 | 0.6926324 | 0.0347277 | 0.5486923 |
| **PWY-5838** | 0.3883765 | 1.274399399 | 0.6307898 | 0.05347034 | 0.689187 | 0.0367715 | 0.5497405 |
| **PWY-5899** | 0.2253542 | 1.228914806 | 0.5964165 | 0.05581835 | 0.689187 | 0.0373196 | 0.5516267 |
| **PWY-7315** | 2.5180414 | 2.97084545 | 0.6203142 | 0.02906066 | 0.6881354 | 0.0446944 | 0.569252 |
| **PWY-6167** | 1.8542966 | -9.804272897 | -0.6935386 | 0.04676508 | 0.6985099 | 0.0341219 | 0.5739645 |
| **PWY-5345** | 2.2296174 | 2.558215162 | 0.6334477 | 0.03477789 | 0.6888053 | 0.0463156 | 0.5822359 |
| **PWY-6148** | 1.1113338 | -9.79849907 | -0.680946 | 0.05237352 | 0.7013925 | 0.0412554 | 0.5860497 |
